# PD-1/PD-L1 inhibitors plus bevacizumab plus chemotherapy versus PD-1/PD-L1 inhibitors plus chemotherapy for advanced non-small cell lung cancer: a phase 3 RCT based meta-analysis

**DOI:** 10.3389/fonc.2025.1496611

**Published:** 2025-05-21

**Authors:** Chao Song, Yuan Qiu, Huan Fan, Yongqing Han

**Affiliations:** ^1^ Department of Thoracic Surgery, The Second Affiliated Hospital, Jiangxi Medical College, Nanchang University, Nanchang, China; ^2^ Department of Oncology, Shangrao People’s Hospital, Shangrao, China; ^3^ Pharmacy Intravenous Admixture Services, The Second Affiliated Hospital, Jiangxi Medical College, Nanchang University, Nanchang, China

**Keywords:** PD-1/PD-L1 inhibitors, bevacizumab, chemotherapy, non-small cell lung cancer, meta-analysis

## Abstract

**Background:**

Combining PD-1/PD-L1 inhibitors with chemotherapy (PIC) is a standard first-line treatment for advanced non-small cell lung cancer (NSCLC). The addition of bevacizumab to this regimen (PD-1/PD-L1 inhibitors+bevacizumab+chemotherapy [PIBC]) remains controversial regarding its potential to enhance antitumor efficacy in clinical practice. This meta-analysis aims to compare the antitumor effectiveness and safety profiles of PIBC with PIC.

**Methods:**

We systematically searched six databases to identify eligible RCTs. The primary outcomes were overall survival (OS) and progression-free survival (PFS), while the secondary outcomes included treatment responses and adverse events (AEs).

**Results:**

Three RCTs (IMpower150, jRCT2080224500, and ORIENT-31) comprising a total of 1529 patients were analyzed. The PIBC regimen significantly improved PFS (hazard ratio [HR]: 0.76 [0.66, 0.87], P < 0.0001), objective response rate (risk ratio [RR]: 1.36 [1.22, 1.51], P < 0.00001), and disease control rate (RR: 1.06 [1.00, 1.12], P = 0.04). The PFS rates were also higher in the PIBC group at 6 and 18 months. Both groups showed similar results in terms of OS, 3–36 month OS rates, and total AEs. However, the PIBC group exhibited a higher incidence of grade 3–5 AEs, serious AEs, grade 3–5 treatment-related AEs (TRAEs) and serious TRAEs. The most frequent grade 3–5 AEs in the PIBC group included anorexia (36.40%), decreased neutrophil count (16.25%), neutropenia (13.50%), reduced white blood cell count (12.12%), and febrile neutropenia (9.42%).

**Conclusions:**

PIBC appears to be better than PIC for advanced NSCLC offering improved PFS and response rates (ORR and DCR). However, its higher incidence of AEs requires cautious attention.

**Systematic review registration:**

https://www.crd.york.ac.uk/PROSPERO/view/CRD42024559146, identifier CRD42024559146.

## Introduction

In recent decades, non-small cell lung cancer (NSCLC) has remained one of the leading cause of incidence and mortality (1). The treatment landscape for advanced NSCLC has significantly evolved in recent years. Chemotherapy was previously the standard treatment for advanced NSCLC, but its limited efficacy often resulted in suboptimal patient outcomes (2). The advent of PD-1/PD-L1 inhibitors has provided new hope, offering improved survival for these patients (3). However, the efficacy of immunotherapy alone has varies across different patient populations (4).

The combination of antiangiogenic agents, such as bevacizumab, with PD-1/PD-L1 inhibitors and chemotherapy (PIBC) shows promise as a treatment strategy for advanced NSCLC. This regimen aims to enhance antitumor efficacy by targeting multiple pathways involved in tumor growth and progression (5). Despite its potential benefits, the clinical advantage of adding bevacizumab to PD-1/PD-L1 inhibitors and chemotherapy remains controversial (6–8). Recent randomized controlled trials (RCTs), including IMpower150, jRCT2080224500, and ORIENT-31, have generated substantial data comparing PIBC with PD-1/PD-L1 inhibitors plus chemotherapy (PIC) (9–11). While these studies provide valuable insights, they also underscore the need for a comprehensive analysis to determine the true clinical value of these regimens.

This meta-analysis systematically reviews and synthesizes data from these RCTs to assess the efficacy and safety of PIBC versus PIC, providing a robust foundation for optimizing treatment protocols in advanced NSCLC.

## Materials and methods

### Search strategy

The search strategy employed keywords such as “PD-1/PD-L1 (See **Supplementary Table S1** for details)”, “Bevacizumab”, “Lung cancer”, and “Randomized”. A comprehensive search was conducted across six databases (PubMed, ScienceDirect, the Cochrane Library, Scopus, EMBASE, and Web of Science) from their inception to March 12, 2025 (**Supplementary Table S1**). Additionally, the reference lists of included studies were examined to identify further eligible RCTs.

### Selection criteria

Inclusion criteria (PICOS):

1. Participants (P): advanced NSCLC.
2. Intervention (I) and control (C): directly comparing PIBC (B includes bevacizumab and its biosimilars) and PIC.
3. Outcomes (O): survival, survival rate, responses, and adverse events (AEs).
4. Study design (S): phase 3 RCTs.

Exclusion criteria: animal experiments, reviews, meta-analyses, case reports and conference articles.

### Data extraction

Data were extracted by two investigators: study characteristics (geographic region, phase, etc.), patient demographics (ECOG PS, TNM Stage, etc.), survival outcomes (overall survival [OS] and progression-free survival [PFS]), survival rates (OS rate [OSR] and PFS rate [PFSR]), responses (objective response rate [ORR], disease control rate [DCR], etc.), and AEs (total, grade 3-5, etc.). Missing data was obtained by contacting the corresponding authors of the included studies. Discrepancies were resolved through re-evaluation.

### Outcome assessments

OS and PFS were subgroup analyzed based on age, sex, race, ECOG PS, smoking status, pathological type, stage, brain metastases, liver metastases, PD-L1 combined positive score (CPS), PD-1/PD-L1 inhibitors type, and epidermal growth factor receptor (EGFR)-mutant.

### Quality assessment

We assessed the quality of RCTs using the Cochrane Risk Assessment Tool and the Jadad scale, which assigns up to 5 points based on randomization, blinding, and participant inclusion. A score of 3 or higher indicates high-quality studies (12, 13). The overall certainty of the evidence was evaluated using the GRADE approach, which considers risk of bias, indirectness, imprecision, and publication bias. This framework categorizes certainty into four levels: very low, low, moderate, and high (14).

### Statistical analysis

The combined data were analyzed using Review Manager 5.3 and STATA 12.0. Survival variables were assessed with the hazard ratio (HR), while dichotomous variables were evaluated with the risk ratio (RR). The OSR was examined at 6–36 months, and the PFSR at 6–24 months. The *I*² statistic and *χ*² test were utilized to evaluate heterogeneity. A fixed-effects model was applied when *I*² was less than 50% or P was greater than 0.1, indicating no notable heterogeneity; otherwise, a random-effects model (This model accounts for both within-study and between-study variability, making it suitable for analyzing data from studies that may have different underlying effect sizes due to variations in study populations, interventions, or other factors.) was used. Statistical significance was defined as P < 0.05. Publication bias was assessed through funnel plot visual inspection. Sensitivity analyses were conducted for primary outcomes (OS, PFS, and ORR) and outcomes with significant heterogeneity. This study adhered to PRISMA guidelines (**Supplementary Table S2**) and was registered in PROSPERO (ID: CRD42024559146).

## Results

### Search results

**Figure 1:**
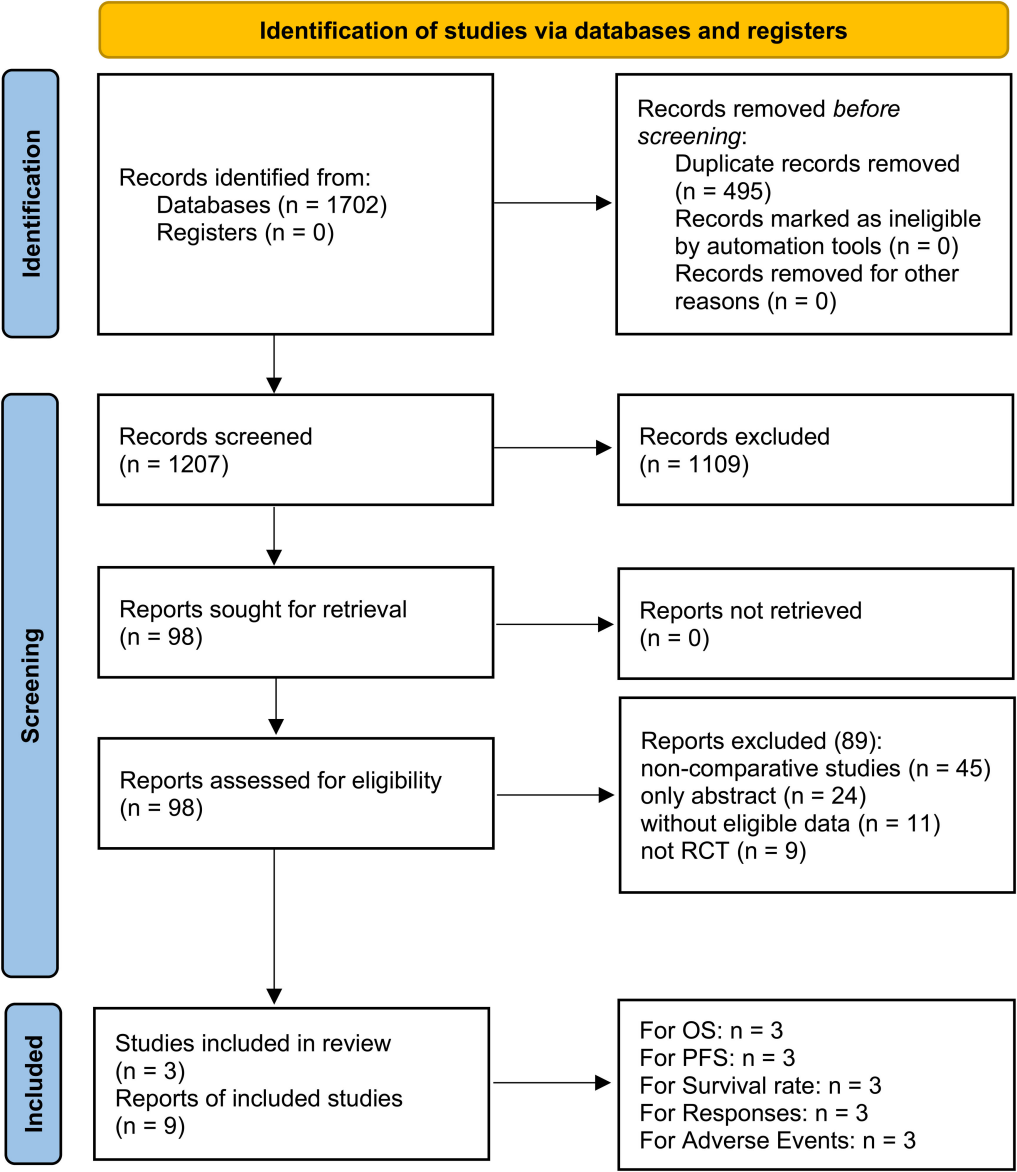
PRISMA flow diagram.

Nine studies derived from three RCTs (IMpower150, jRCT2080224500, and ORIENT-31) were included, comprising 763/766 patients in the PIBC/PIC groups (**Figure 1**) (9–11, 15–20). IMpower150 is a global multicenter study, whereas jRCT2080224500 and ORIENT-31 were conducted in Asia. All three studies were classified as high quality (**Supplementary Figure S1**, **Supplementary Table S3**). According to the GRADE approach, the certainty of evidence ranged from moderate to high (**Supplementary Table S4**). **Table 1** provides a summary of the baseline information of the included RCTs.

**Table 1:** Baseline characteristics of the included studies.

Study	Phase	Country	Groups	Patients	Sex (M/F)	Age (Mean, year)	ECOG PS		Smoking status		TNM Stage		PD-1/PD-L1 type	Follow up (months)	Quality (score)
							0	1	III	IV	III	IV			
IMpower150 (NCT02366143, 2015.03-2016.12)															
Nogami 2022 (15), West 2022 (16), Socinski 2021 (17), Reck 2020 (18), Reck 2019 (19), Socinski 2018 (9)	III	Global multicenter	PIBC	40	240/160	63	159	238	318	82	0	400	Atezolizumab	39.4	5
			PIC	402	241/161	63	180	222	325	77	0	402			
jRCT2080224500 (2019.01-2020.08)															
Shiraishi 2024 (10)	III	Japan	PIBC	205	132/73	68	98	107	148	57	6	199	Atezolizumab	24	5
			PIC	206	141/65	67	92	114	148	58	3	203			
ORIENT-31 (NCT03802240, 2019.07-2022.03)															
Lu 2023 (20), Lu 2022 (11)	III	China	PIBC	158	65/93	58.5	38	120	47	111	6	152	Sintilimab	15.1	5
			PIC	158	65/93	57.5	22	136	49	109	5	153			

ECOG PS, Eastern Cooperative Oncology Group Performance Status; M/F, Male/Female; PD-1, Programmed cell death protein 1; PD-L1, Programmed cell death 1 ligand 1; PIBC, PD-1/PD-L1 Inhibitors plus Bevacizumab plus chemotherapy; PIC, PD-1/PD-L1 Inhibitors plus chemotherapy; TNM, Tumor Node Metastasis.

### Survival

**Figure 2:**
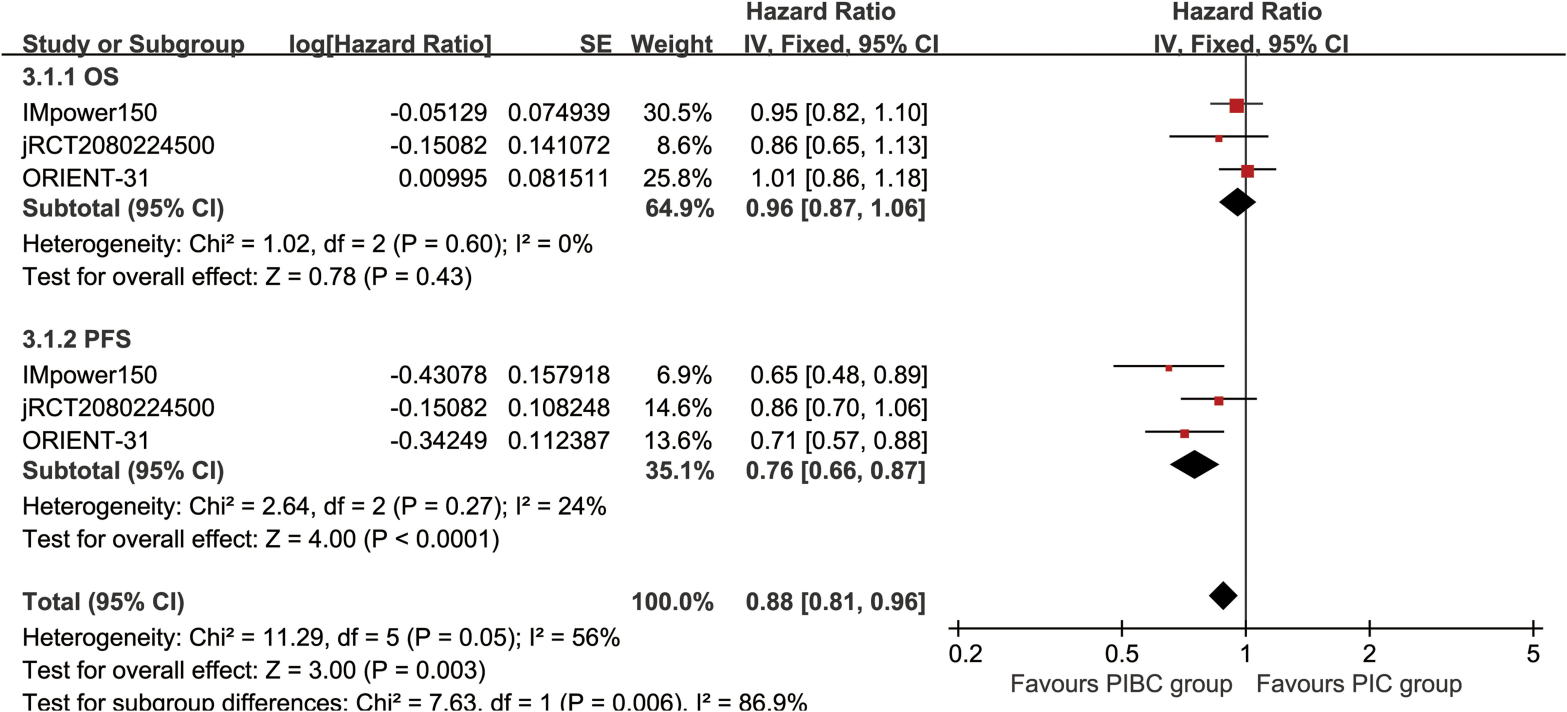
Forest plots of overall survival and progression-free survival associated with PIBC versus PIC.

**Figure 3:**
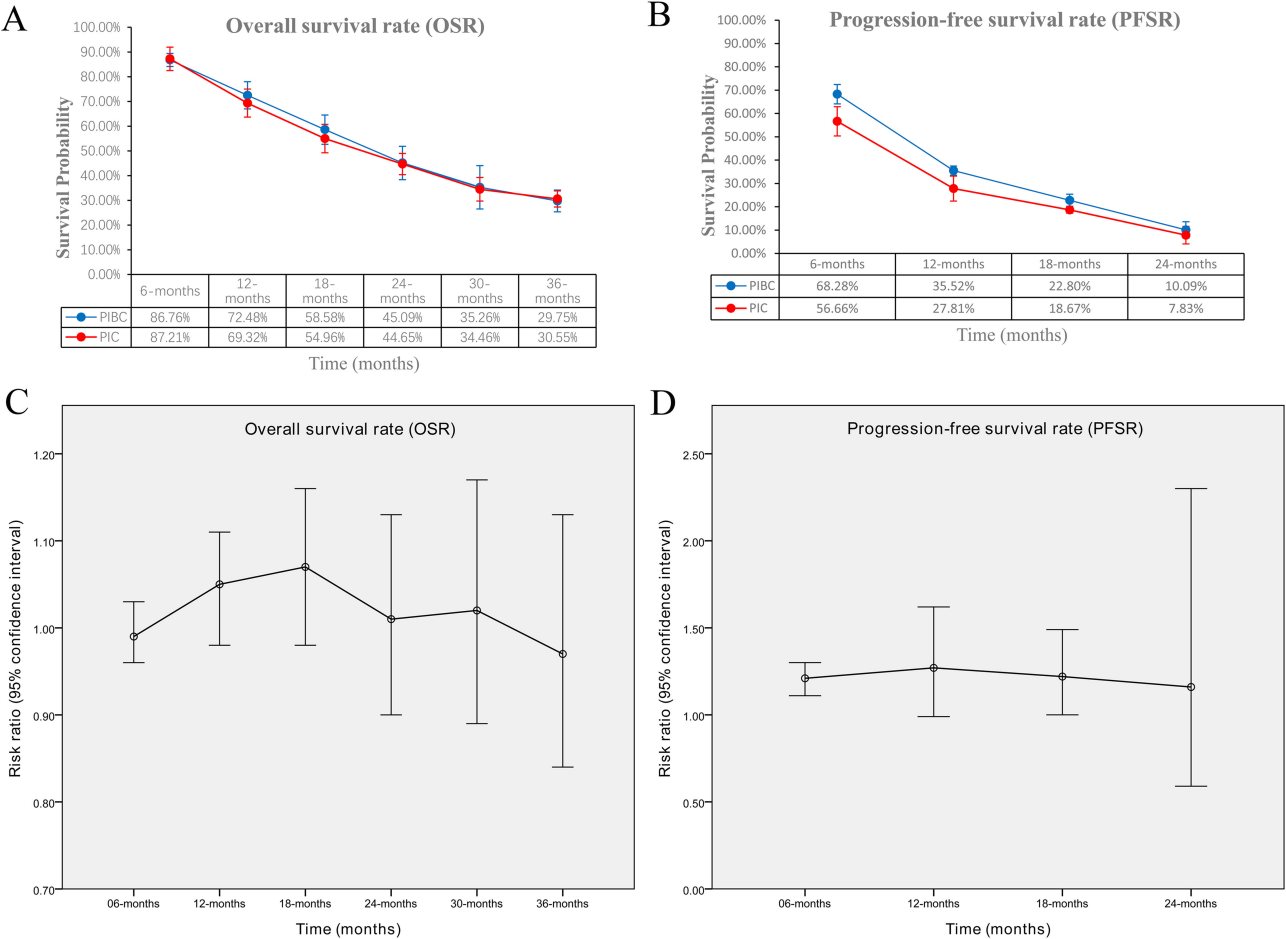
Comparisons of OSR and PFSR. **(A)** OSR at 3–36 months between the two groups; **(B)** PFSR at 3–24 months between the two groups; **(C)** trend of risk ratios in OSR; **(D)** trend of risk ratios in PFSR.

OS was comparable between the two groups (HR: 0.96 [0.87, 1.06], P = 0.43) (**Figure 2**). The OSR at 6–36 months showed no significant difference between two groups (**Supplementary Figure S2**). Detailed comparisons of OSR and its temporal changes over time are presented in **Figures 3A, C**. Older age may be a favorable factor for the PIBC group (**Supplementary Table S5**).

PFS was significantly better in the PIBC group (HR: 0.76 [0.66, 0.87], P < 0.0001) (**Figure 2**). The PFSRs were notably higher in the PIBC group at both 6 months (HR: 1.21 [1.11, 1.30], P < 0.00001) and 18 months (HR: 1.22 [1.00, 1.49], P = 0.05). (**Supplementary Figure S3**). Detailed comparisons of PFSR and its changes over time are presented in **Figures 3B, D**. Sex- female, smoking status-never, PD-L1 CPS <1%, and driver gene alterations-positive may be favorable factors for the PIC group (**Supplementary Table S5**).

### Responses

**Figure 4:**
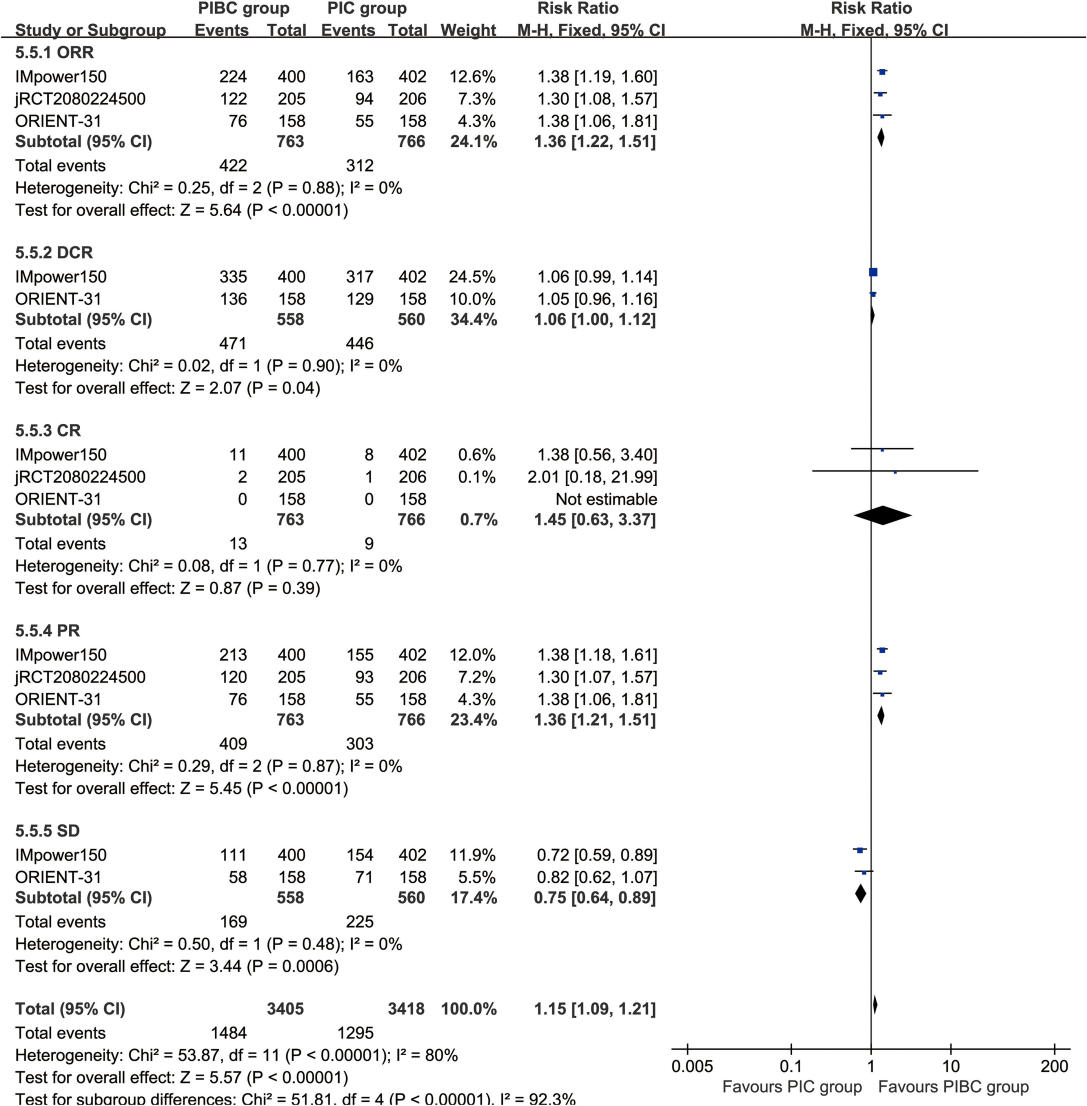
Forest plots of responses associated with PIBC versus PIC.

The ORR (RR: 1.36 [1.22, 1.51], P < 0.00001), DCR (RR: 1.06 [1.00, 1.12], P = 0.04), and partial response [PR] (RR: 1.36 [1.21, 1.51], P < 0.00001) were significantly higher in the PIBC group. Although the CR (RR: 1.45 [0.63, 3.37], P = 0.39) favored the PIBC group, the difference was not statistically significant. Conversely, the rate of stable disease [SD] (RR: 0.75 [0.64, 0.89], P = 0.0006) was higher in the PIC group (**Figure 4**).

### Safety

In summary, the rates of grade 3–5 AEs (RR: 1.10 [1.01, 1.19], P = 0.03), fatal AEs (RR: 2.70 [1.45, 5.05], P = 0.002), discontinuations due to AEs (RR: 2.58 [2.03, 3.28], P < 0.00001), dose interruptions due to AEs (RR: 1.23 [1.09, 1.39], P = 0.0006), grade 3–5 treatment-related AEs (TRAEs) (RR: 1.33 [1.18, 1.50], P < 0.00001), serious TRAEs (RR: 1.36 [1.10, 1.69], P = 0.004), and fatal TRAEs (RR: 3.85 [1.58, 9.40], P = 0.003) were significantly higher in the PIBC group. Total AEs, serious AEs, and total TRAEs showed no significant difference between two groups (**Table 2**, **Supplementary Figure S4**).

**Table 2:** Summary of adverse events.

Adverse events	PIBC		PIC		Risk ratio [95% CI]	P
	Event/total	%	Event/total	%		
Total adverse events	747/763	97.90%	745/766	97.26%	1.01 [0.99, 1.02]	0.41
Grade 3–5 adverse events	465/763	60.94%	426/766	55.61%	1.10 [1.01, 1.19]	0.03
Serious adverse events	252/558	45.16%	210/560	37.50%	1.29 [0.91, 1.82]	0.15
Fatal adverse events	35/558	6.27%	13/560	2.32%	2.70 [1.45, 5.05]	0.002
Discontinuation due to adverse events	190/558	34.05%	74/560	13.21%	2.58 [2.03, 3.28]	<0.00001
Dose interruption due to adverse events	256/400	64.00%	209/402	51.99%	1.23 [1.09, 1.39]	0.0006
Treatment-related adverse events	526/558	94.27%	528/560	94.29%	1.01 [0.96, 1.06]	0.71
Grade 3–5 treatment-related adverse events	313/558	56.09%	236/560	42.14%	1.33 [1.18, 1.50]	<0.00001
Serious treatment-related adverse events	155/558	27.78%	114/560	20.36%	1.36 [1.10, 1.69]	0.004
Fatal treatment-related adverse events	23/763	3.01%	6/766	0.78%	3.85 [1.58, 9.40]	0.003

CI, confidence interval; PD-1, Programmed cell death protein 1; PD-L1, Programmed cell death 1 ligand 1; PIBC, PD-1/PD-L1 Inhibitors plus Bevacizumab plus chemotherapy; PIC, PD-1/PD-L1 Inhibitors plus chemotherapy.

Regarding any grade AEs, the PIBC group exhibited higher rates of anorexia, nausea, malaise, peripheral neuropathy, decreased appetite, elevated creatinine, stomatitis, increased blood thyroid stimulating hormone, vomiting, elevated γ-glutamyltransferase, diarrhea, dry skin, elevated amylase, headache, back pain, and febrile neutropenia (**Table 3**, **Supplementary Table S5**).

**Table 3:** Any grade adverse events (incidence rate > 10% in the PIBC group).

Adverse events	PIBC		PIC		Risk ratio [95% CI]	P
	Event/total	%	Event/total	%		
Anorexia	98/205	47.80%	74/206	35.92%	1.33 [1.06, 1.68]	0.02
Alopecia	183/400	45.75%	173/402	43.03%	1.06 [0.91, 1.24]	0.44
Nausea	320/763	41.94%	268/766	34.99%	1.20 [1.06, 1.36]	0.005
Malaise	85/205	41.46%	56/206	27.18%	1.53 [1.16, 2.01]	0.003
White blood cell count decreased	146/363	40.22%	168/364	46.15%	0.87 [0.74, 1.02]	0.09
AST increased	138/363	38.02%	114/364	31.32%	1.21 [0.99, 1.48]	0.06
Peripheral neuropathy	152/400	38.00%	122/402	30.35%	1.25 [1.03, 1.52]	0.02
Anemia	269/763	35.26%	284/766	37.08%	0.95 [0.84, 1.08]	0.42
ALT increased	126/363	34.71%	109/364	29.95%	1.15 [0.81, 1.64]	0.43
Fever	71/205	34.63%	67/206	32.52%	1.06 [0.81, 1.40]	0.65
Neutrophil count decreased	250/763	32.77%	232/766	30.29%	1.08 [0.95, 1.23]	0.24
Decreased appetite	168/558	30.11%	132/560	23.57%	1.28 [1.06, 1.54]	0.01
Constipation	203/763	26.61%	204/766	26.63%	1.01 [0.71, 1.43]	0.99
Fatigue	101/400	25.25%	89/402	22.14%	1.14 [0.89, 1.46]	0.3
Hypertension	191/763	25.03%	90/766	11.75%	3.06 [0.91, 10.33]	0.07
Asthenia	133/558	23.84%	127/560	22.68%	1.05 [0.86, 1.29]	0.64
Platelet count decreased	180/763	23.59%	158/766	20.63%	1.14 [0.95, 1.37]	0.15
Creatinine increased	73/363	20.11%	52/364	14.29%	1.41 [1.02, 1.94]	0.04
Proteinuria	149/763	19.53%	76/766	9.92%	2.31 [0.86, 6.15]	0.1
Stomatitis	118/605	19.50%	57/608	9.38%	2.08 [1.56, 2.77]	<0.00001
Increased blood thyroid stimulating hormone	29/158	18.35%	16/158	10.13%	1.81 [1.03, 3.20]	0.04
Neutropenia	72/400	18.00%	68/402	16.92%	1.06 [0.79, 1.44]	0.69
Vomiting	137/763	17.96%	107/766	13.97%	1.28 [1.02, 1.61]	0.03
Weight decreased	28/158	17.72%	25/158	15.82%	1.12 [0.68, 1.83]	0.65
Epistaxis	107/605	17.69%	70/608	11.51%	4.38 [0.08, 241.52]	0.47
Arthralgia	66/400	16.50%	59/402	14.68%	1.12 [0.81, 1.55]	0.48
γ-glutamyltransferase increased	59/363	16.25%	30/364	8.24%	1.97 [1.31, 2.97]	0.001
Diarrhea	123/763	16.12%	91/766	11.88%	1.36 [1.06, 1.74]	0.02
Hypothyroidism	24/158	15.19%	17/158	10.76%	1.41 [0.79, 2.52]	0.24
Dry skin	31/205	15.12%	18/206	8.74%	1.73 [1.00, 2.99]	0.05
Hiccups	31/205	15.12%	27/206	13.11%	1.15 [0.72, 1.86]	0.56
Increased amylase	52/363	14.33%	23/364	6.32%	2.27 [1.42, 3.62]	0.0006
Headache	28/205	13.66%	9/206	4.37%	3.13 [1.51, 6.46]	0.002
Peripheral edema	28/205	13.66%	33/206	16.02%	0.85 [0.54, 1.36]	0.5
Myalgia	53/400	13.25%	47/402	11.69%	1.13 [0.78, 1.64]	0.5
Thrombocytopenia	52/400	13.00%	45/402	11.19%	1.16 [0.80, 1.69]	0.43
Rash maculopapular	25/205	12.20%	16/206	7.77%	1.57 [0.86, 2.85]	0.14
Rash	92/763	12.06%	66/766	8.62%	1.29 [0.60, 2.78]	0.52
Insomnia	23/205	11.22%	33/206	16.02%	0.70 [0.43, 1.15]	0.16
Back pain	23/205	11.22%	9/206	4.37%	2.57 [1.22, 5.41]	0.01
Lymphocyte count decreased	17/158	10.76%	16/158	10.13%	1.06 [0.56, 2.03]	0.85
Blood lactate dehydrogenase increased	17/158	10.76%	18/158	11.39%	0.94 [0.51, 1.76]	0.86
Paresthesia	42/400	10.50%	37/402	9.20%	1.14 [0.75, 1.74]	0.54

ALT, Alanine Aminotransferase; AST, Aspartate Aminotransferase; CI, confidence interval; PD-1, Programmed cell death protein 1; PD-L1, Programmed cell death 1 ligand 1; PIBC, PD-1/PD-L1 Inhibitors plus Bevacizumab plus chemotherapy; PIC, PD-1/PD-L1 Inhibitors plus chemotherapy.

For grade 3–5 AEs, the PIBC group had more instances of grade 3–5 anorexia, febrile neutropenia, elevated ALT, increased γ-glutamyltransferase, and decreased appetite (**Table 4**, **Supplementary Table S6**).

**Table 4:** Grade 3–5 adverse events (incidence rate > 2% in the PIBC group).

Adverse events	PIBC		PIC		Risk ratio [95% CI]	P
	Event/total	%	Event/total	%		
Anorexia	74/205	36.10%	12/206	5.83%	6.20 [3.47, 11.05]	<0.00001
Neutrophil count decreased	124/763	16.25%	109/766	14.23%	1.14 [0.91, 1.44]	0.26
Neutropenia	54/400	13.50%	44/402	10.95%	1.23 [0.85, 1.79]	0.27
White blood cell count decreased	44/363	12.12%	42/364	0.12	1.05 [0.71, 1.56]	0.81
Febrile neutropenia	57/605	9.42%	36/608	5.92%	1.59 [1.06, 2.38]	0.02
Anemia	67/763	8.78%	71/766	0.09	1.01 [0.56, 1.80]	0.99
Hypertension	62/763	8.13%	31/766	4.05%	3.16 [0.75, 13.36]	0.12
Platelet count decreased	58/763	7.60%	40/766	5.22%	1.46 [0.99, 2.14]	0.06
ALT increased	17/363	4.68%	5/364	1.37%	3.20 [1.25, 8.20]	0.02
Thrombocytopenia	16/400	4.00%	17/402	4.23%	0.95 [0.48, 1.85]	0.87
γ-glutamyltransferase increased	14/363	3.86%	3/364	0.82%	4.68 [1.36, 16.15]	0.01
Fatigue	13/400	3.25%	10/402	0.02	1.31 [0.58, 2.94]	0.52
Myelosuppression	5/158	3.16%	1/158	0.63%	5.00 [0.59, 42.31]	0.14
Lymphocyte count decreased	5/158	3.16%	2/158	1.27%	2.50 [0.49, 12.70]	0.27
Nausea	24/763	3.15%	18/766	0.02	1.34 [0.73, 2.45]	0.34
Decreased appetite	16/558	2.87%	3/560	0.01	4.73 [1.50, 14.92]	0.01
Pneumonitis	10/363	2.75%	9/364	2.47%	1.12 [0.46, 2.71]	0.81
Peripheral neuropathy	11/400	2.75%	9/402	2.24%	1.23 [0.51, 2.93]	0.64
Increased amylase	9/363	2.48%	4/364	1.10%	2.26 [0.70, 7.25]	0.17
Proteinuria	17/763	2.23%	11/766	1.44%	1.51 [0.73, 3.11]	0.27

ALT, Alanine Aminotransferase; AST, Aspartate Aminotransferase; CI, confidence interval; PD-1, Programmed cell death protein 1; PD-L1, Programmed cell death 1 ligand 1; PIBC, PD-1/PD-L1 Inhibitors plus Bevacizumab plus chemotherapy; PIC, PD-1/PD-L1 Inhibitors plus chemotherapy.

### Sensitivity analysis

Sensitivity analyses for PFSR-12m, rash, and anemia were conducted, demonstrated that omitting any individual study did not alter the results’ reliability (**Supplementary Figure S5**). Similarly, for the main outcomes (OS, PFS, and ORR), omitting any individual study also did not alter the results’ reliability (**Supplementary Figure S6**).

### Publication bias

**Figure 5:**
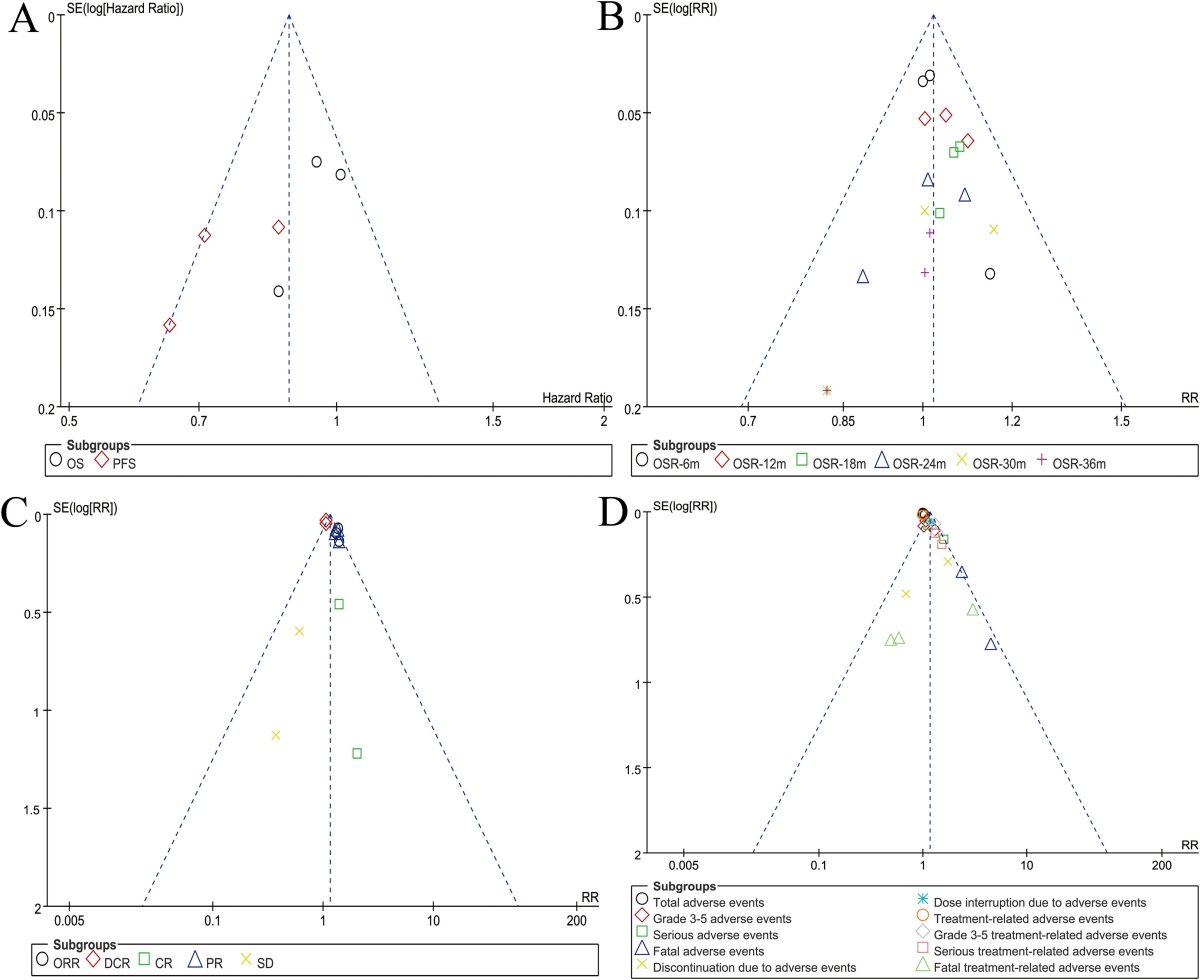
Funnel plots of survival **(A)**, OSR **(B)**, responses **(C)**, and safety summary **(D)**.

Symmetry in funnel plots for survival, OSR, responses, and safety summary suggested an acceptable level of publication bias (**Figure 5**).

## Discussion

PIC is a common first-line treatment for NSCLC without driver gene mutations or for NSCLC with driver gene mutations that have developed resistance to targeted therapy (9–11). However, the addition of bevacizumab (PIBC) to the regimen remains controversial regarding its potential to enhance antitumor efficacy in clinical practice. The ATTLAS study demonstrated that adding atezolizumab and bevacizumab to chemotherapy significantly improved progression-free survival and objective response rates in EGFR- or ALK-mutated NSCLC patients who had progressed after tyrosine kinase inhibitor therapy (21). Similarly, the IMpower151 trial compared atezolizumab plus bevacizumab and chemotherapy with bevacizumab and chemotherapy alone in the first-line treatment of metastatic nonsquamous NSCLC, further supporting the potential of multi-agent immunotherapy regimens (22). However, both the ATTLAS and IMpower151 studies were excluded because they did not include a PIC arm, which is essential for directly addressing our research question regarding the additive value of bevacizumab. Ultimately, our meta-analysis directly compared the efficacy and safety of PIBC versus PIC using data from three phase 3 RCTs (IMpower150, jRCT2080224500, and ORIENT-31) (9–11). The results indicated that the PIBC regimen significantly improved PFS, ORR, and DCR. Additionally, the PFS rates at 6 and 18 months were higher in the PIBC group. Both groups were similar in terms of OS, OS rates at 3–36 months, and total AEs. However, the PIBC group exhibited higher rates of grade 3–5 TRAEs, serious TRAEs, and fatal TRAEs.

The primary advantage of the PIBC regimen lies in its superior PFS, a finding consistent with IMpower150 and ORIENT-31 (9, 11). The ORR and DCR were also notably higher in the PIBC group. These findings suggest a more robust tumor response and stabilization, consistent with the enhanced anti-angiogenic and immune-modulatory effects of the combination therapy (23, 24). The addition of bevacizumab appears to potentiate PD-1/PD-L1 inhibitors by normalizing tumor vasculature, improving immune cell infiltration, and enhancing chemotherapy delivery (25, 26). Despite these improvements in PFS and ORR, the OS benefit was less pronounced. These findings are further supported by a recent retrospective cohort study by Yang et al. (2024), which evaluated the efficacy and safety of PIBC regimen in 65 patients with driver gene-negative advanced-stage lung adenocarcinoma (27). Their results showed a significantly improved median PFS in the PIBC group compared to the bevacizumab plus chemotherapy (BC) group, while OS showed a non-significant trend in favor of PBC (20.6 vs. 15.9 months; P = 0.115). Multivariate Cox regression confirmed the PIBC regimen as an independent factor for prolonged PFS. However, no statistically significant OS benefit was observed, consistent with our meta-analysis findings. This discrepancy between PFS and OS may be explained by several factors. Firstly, subsequent lines of therapy post-progression may influence OS outcomes, as patients who progress on one therapy often receive additional treatments that can affect survival (28). Secondly, the development of resistance mechanisms, such as upregulation of alternative growth pathways or immune evasion strategies, may mitigate the long-term benefits of the initial treatment (29, 30). Furthermore, the aggressive nature of advanced NSCLC, particularly in patients with high tumor burden or poor performance status, may limit the potential for OS improvement despite initial PFS gains (31). The heterogeneity of patient populations across different studies also contributes to the varied survival outcomes. Differences in PD-L1 expression, and other molecular characteristics can influence response to therapy and long-term survival (32, 33). Patients with high PD-L1 expression may experience enhanced benefits from PIBC therapy due to more effective immune modulation and improved response rates. Conversely, patients with lower PD-L1 expression may not achieve the same level of efficacy, potentially requiring alternative therapeutic approaches (34, 35). Moreover, tumor stage at diagnosis can influence outcomes, with more advanced stages potentially showing variable responses due to differences in tumor burden and microenvironment (36). The addition of bevacizumab may enhance the effectiveness of PD-1/PD-L1 inhibitors by normalizing tumor vasculature, thereby improving drug delivery and immune cell infiltration in the tumor microenvironment (37). However, this combination may also increase the risk of severe adverse events, particularly in patients with pre-existing inflammatory conditions or compromised organ function (38). The dual impact of immune modulation and vascular normalization highlights the need for careful patient selection to maximize benefits while minimizing risks (39). Integrating molecular profiling and biomarker-driven strategies into clinical practice could further refine patient selection and optimize treatment outcomes.

While PIBC offers promising efficacy, it raises significant safety concerns. The PIBC also raises significant safety concerns, particularly regarding severe TRAEs. Grade 3–5 AEs were notably more prevalent in the PIBC group, including anorexia, febrile neutropenia, ALT increased, hypertension, proteinuria, and hemorrhage, in line with bevacizumab’s established safety profile (40). The heightened risk of hypertension is particularly concerning, necessitating careful monitoring and management to prevent cardiovascular complications. Proteinuria and hemorrhage also warrant close surveillance through regular renal function tests and bleeding assessments (41, 42). Managing these AEs requires a multidisciplinary approach, including routine monitoring, early symptom detection, and timely intervention (43, 44). Prophylactic measures, such as the use of granulocyte colony-stimulating factors (G-CSFs) to manage neutropenia, are essential to minimize treatment disruptions and maintain dose intensity (45, 46). The potential for severe TRAEs underscores the need for comprehensive patient education and the implementation of rapid-response protocols to manage complications effectively (47). Patient selection is crucial when considering PIBC. Factors such as performance status, prior treatment history, and comorbid conditions should be evaluated to balance the benefits of extended PFS against the risks of severe TRAEs. Personalized treatment plans, informed by biomarkers like PD-L1 expression, can optimize therapeutic outcomes (48, 49). Additionally, further clinical trials are needed to refine the safety profile of PIBC and develop strategies to mitigate AEs. For instance, prophylactic antihypertensive medications or bevacizumab dose modifications may help manage TRAEs more effectively (50, 51).

Our findings offer important insights into the comparative efficacy and safety of PIBC versus PIC; however, several limitations should be acknowledged, and potential solutions should be considered to improve future research in this field. First, restricting the analysis to English-language articles introduces language bias. Future meta-analyses should consider incorporating studies in multiple languages, potentially with professional translation support, to reduce potential selection bias. Second, the inclusion of only three RCTs limits the generalizability of our findings. Expanding the analysis by incorporating ongoing or recently completed RCTs could provide a more comprehensive and up-to-date evaluation of PIBC versus PIC. Third, the lack of individual patient data (IPD) precluded an IPD meta-analysis, which could have enhanced clinical relevance. Fourth, our study only included trials evaluating Atezolizumab and Sintilimab, so the results may not be representative of other PD-1/PD-L1 inhibitors. Further meta-analyses incorporating data from trials evaluating other PD-1/PD-L1 inhibitors, such as pembrolizumab, nivolumab, and durvalumab, are necessary to validate our findings. Fifth, the predominance of Asian patients in the included studies may limit the applicability of the results to other populations. Future studies should aim to incorporate data from more diverse geographic regions to enhance the external validity of the findings. Sixth, none of the included RCTs reported quality of life outcomes, which are essential for interpreting the real-world impact of treatments, especially when differences in toxicity profiles are observed. This represents an important gap in the current evidence and should be a key focus in future clinical research on PIBC regimens.

## Conclusion

PIBC appears to be superior to PIC for advanced NSCLC offering improved PFS and higher response rates (ORR and DCR). However, OS and OSR at 6 to 36 months were comparable between the two groups. The increased risk of severe AEs necessitates cautious use and proactive management. Further research, including IPD meta-analyses, large multi-regional trials, and biomarker-driven studies, is needed to refine patient selection, identify predictive biomarkers, and develop strategies to mitigate adverse effects.

## Data Availability

The original contributions presented in the study are included in the article/**Supplementary Material**. Further inquiries can be directed to the corresponding author.

## Supplementary material

Supplementary Figure 1 Cochrane risk assessment.

Supplementary Figure 2 Forest plots of OSR at 6–36 months associated with PIBC versus PIC.

Supplementary Figure 3 Forest plots of PFSR at 6–24 months associated with PIBC versus PIC.

Supplementary Figure 4 Forest plots of safety summary associated with PIBC versus PIC.

Supplementary Figure 5 Sensitivity analysis of PFSR-12m **(A)**, rash **(B)**, and anemia **(C)**.

Supplementary Figure 6 Sensitivity analysis of OS **(A)**, PFS **(B)**, and ORR **(C)**.
