# Supplementary figures and images for: PD-1/PD-L1 inhibitors plus bevacizumab plus chemotherapy versus PD-1/PD-L1 inhibitors plus chemotherapy for advanced non-small cell lung cancer: a phase 3 RCT based meta-analysis
Source: Front Oncol. 2025 May 21;15:1496611. doi: 10.3389/fonc.2025.1496611 (PMC12133818; doi:10.3389/fonc.2025.1496611)

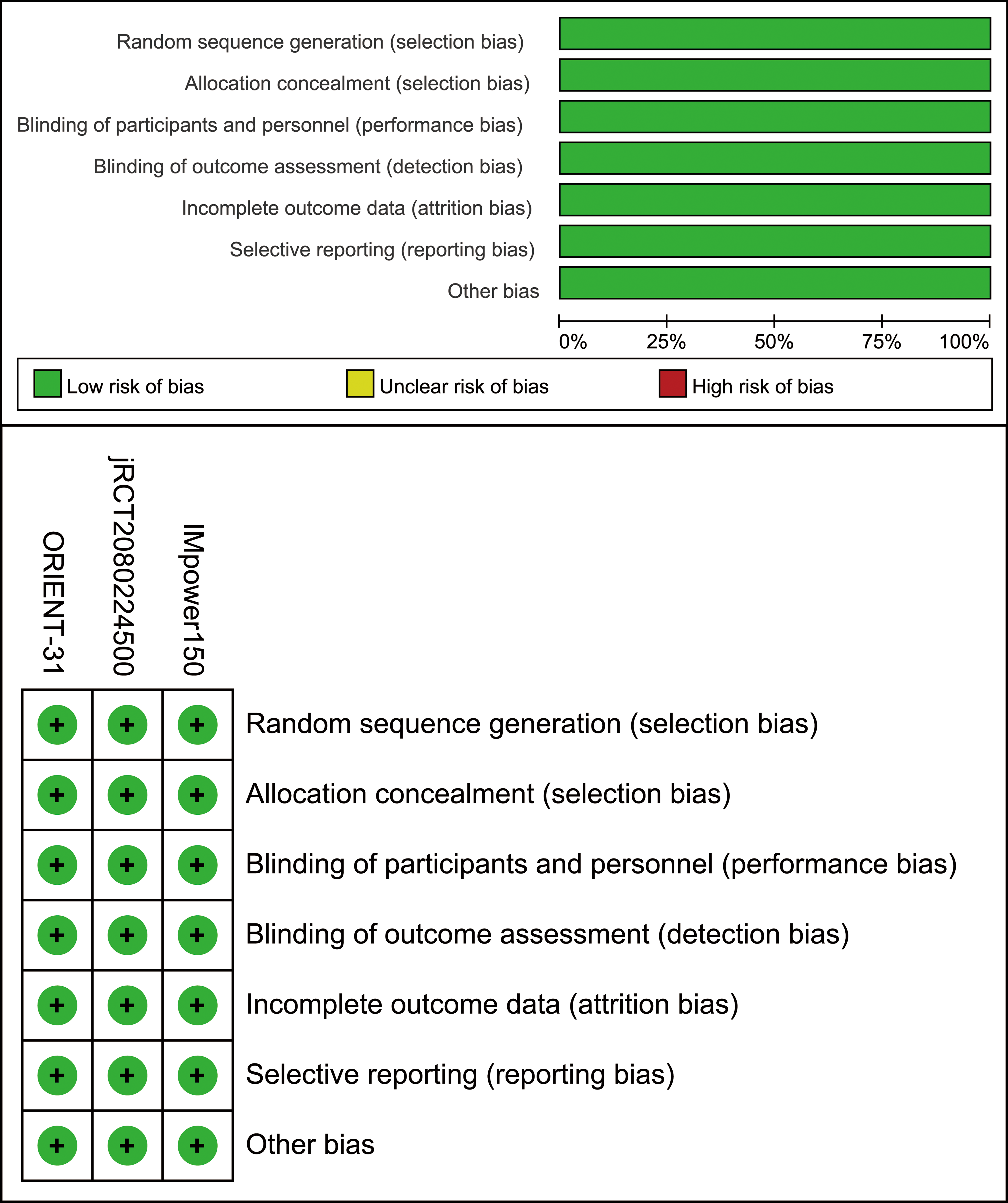

Supplement: Supplementary Figure 1 — Cochrane risk assessment. [file Image1.tif]

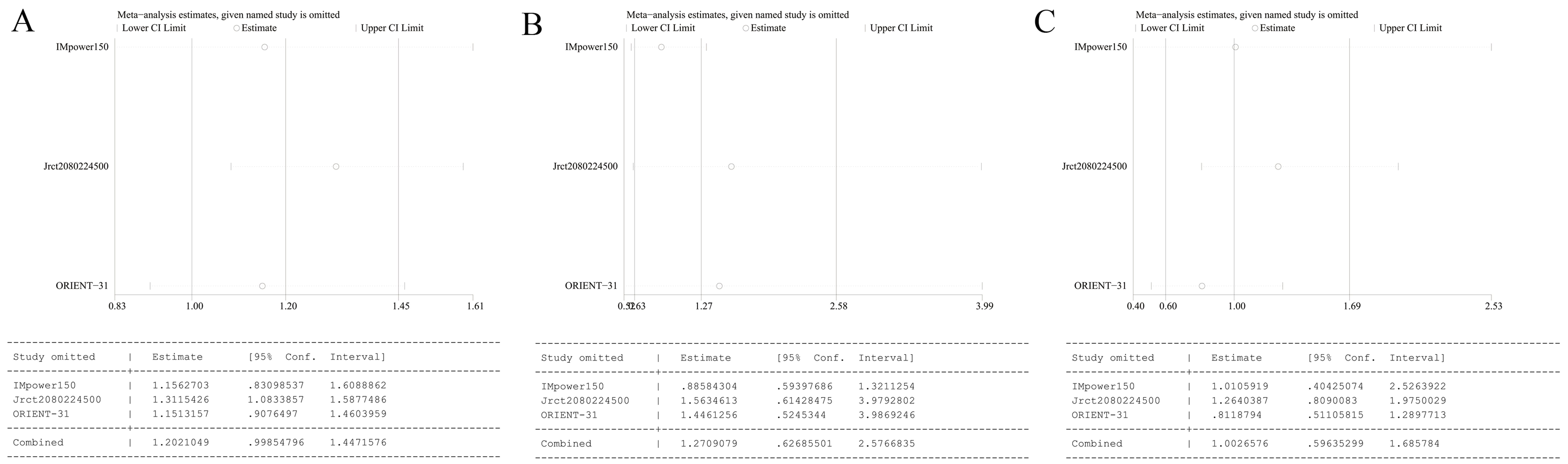

Supplement: Supplementary Figure 5 — Sensitivity analysis of PFSR-12m (A), rash (B), and anemia (C). [file Image5.tif]

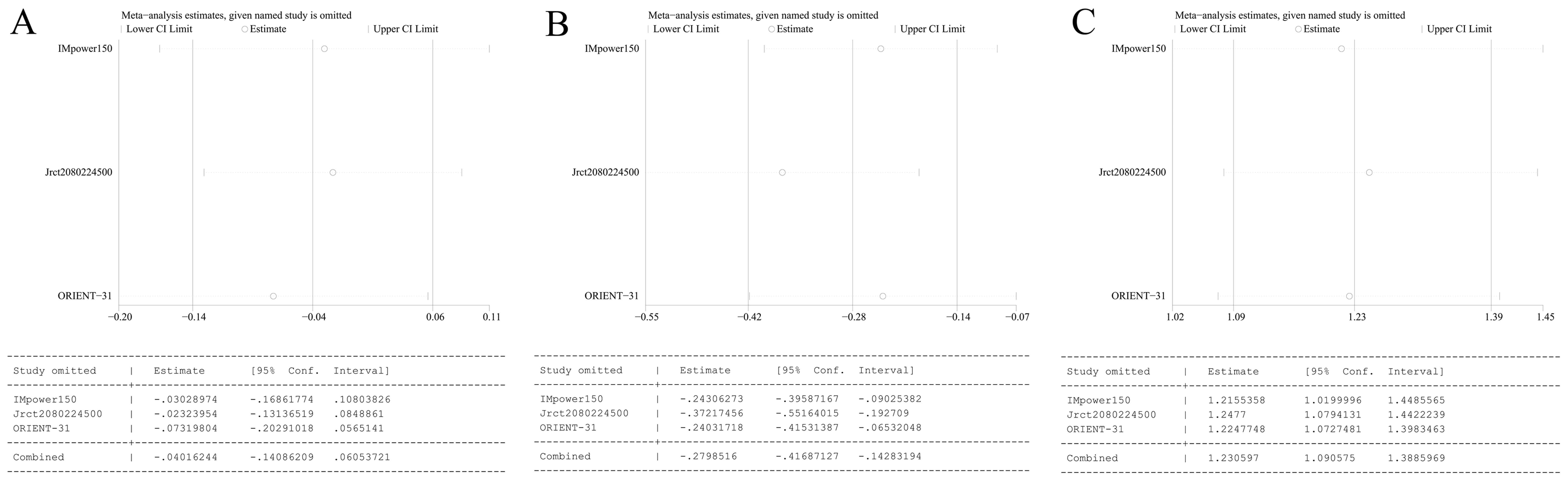

Supplement: Supplementary Figure 6 — Sensitivity analysis of OS (A), PFS (B), and ORR (C). [file Image6.tif]
