# Supplementary material for: PD-1/PD-L1 inhibitors plus bevacizumab plus chemotherapy versus PD-1/PD-L1 inhibitors plus chemotherapy for advanced non-small cell lung cancer: a phase 3 RCT based meta-analysis
Source: Front Oncol. 2025 May 21;15:1496611. doi: 10.3389/fonc.2025.1496611 (PMC12133818; doi:10.3389/fonc.2025.1496611)
Supplement: Supplementary file 9 [file Table3.doc]

**Table S3 Methodological quality assessments (Jadad scale) of the included studies.**

| **Study** | | **Randomization** | **Masking** | **Accountability of all patients** | **Quality (score)** |
| --- | --- | --- | --- | --- | --- |
| IMpower150 (NCT02366143) | West 2022[15], Nogami 2022[16], Socinski 2021[17], Reck 2020[18], Reck 2019[19], Socinski 2018[9] | ** | ** | * | 5 |
| jRCT2080224500 | Shiraishi 2024[10] | ** | ** | * | 5 |
| ORIENT-31 (NCT03802240) | Lu 2023[20], Lu 2022[11] | ** | ** | * | 5 |
