# Supplementary material for: PD-1/PD-L1 inhibitors plus bevacizumab plus chemotherapy versus PD-1/PD-L1 inhibitors plus chemotherapy for advanced non-small cell lung cancer: a phase 3 RCT based meta-analysis
Source: Front Oncol. 2025 May 21;15:1496611. doi: 10.3389/fonc.2025.1496611 (PMC12133818; doi:10.3389/fonc.2025.1496611)
Supplement: Supplementary file 10 [file Table4.doc]

**Table S4** GRADE quality assessment by therapeutic strategy and study design for the outcomes.

| **Outcomes** | **No. of Participants** | | **Differences (95%CI) ^a^** | **Quality Assessment** | | | | | **Quality** |
| --- | --- | --- | --- | --- | --- | --- | --- | --- | --- |
|  | **PIBC** | **PIC** |  | **Risk of Bias^b^** | **Inconsistency** | **Indirectness** | **Imprecision** | **Publication Bias^c^** |  |
| **Survival** |  |  |  |  |  |  |  |  |  |
| OS | 763 | 766 | 0.96 [0.87, 1.06] | Low | No inconsistency | No indirectness | No imprecision | Unlikely | High |
| PFS | 763 | 766 | 0.76 [0.66, 0.87] | Low | No inconsistency | No indirectness | No imprecision | Unlikely | High |
| **Survival rate** |  |  |  |  |  |  |  |  |  |
| **OSR** |  |  |  |  |  |  |  |  |  |
| OSR-6m | 662/763 | 668/766 | 0.99 [0.96, 1.03] | Low | No inconsistency | No indirectness | No imprecision | Unlikely | High |
| OSR-12m | 553/763 | 531/766 | 1.05 [0.98, 1.11] | Low | No inconsistency | No indirectness | No imprecision | Unlikely | High |
| OSR-18m | 447/763 | 421/766 | 1.07 [0.98, 1.16] | Low | No inconsistency | No indirectness | No imprecision | Unlikely | High |
| OSR-24m | 344/763 | 342/766 | 1.01 [0.90, 1.13] | Low | No inconsistency | No indirectness | No imprecision | Unlikely | High |
| OSR-30m | 269/763 | 264/766 | 1.02 [0.89, 1.17] | Low | No inconsistency | No indirectness | No imprecision | Unlikely | High |
| OSR-36m | 227/763 | 234/766 | 0.97 [0.84, 1.13] | Low | No inconsistency | No indirectness | No imprecision | Unlikely | High |
| **PFSR** |  |  |  |  |  |  |  |  |  |
| PFSR-6m | 521/763 | 434/766 | 1.21 [1.11, 1.30] | Low | No inconsistency | No indirectness | No imprecision | Unlikely | High |
| PFSR-12m | 271/763 | 213/766 | 1.27 [0.99, 1.62] | Low | Serious (-1) | No indirectness | No imprecision | Unlikely | Medium |
| PFSR-18m | 174/763 | 143/766 | 1.22 [1.00, 1.49] | Low | No inconsistency | No indirectness | No imprecision | Unlikely | High |
| PFSR-24m | 77/763 | 60/766 | 1.16 [0.59, 2.30] | Low | Serious (-1) | No indirectness | No imprecision | Unlikely | Medium |
| Subgroup analysis of OS |  |  |  |  |  |  |  |  |  |
| Total | 1529 | | 0.96 [0.87, 1.06] | Low | No inconsistency | No indirectness | No imprecision | Unlikely | High |
| Age - < 75 years | 356 | | 0.91 [0.68, 1.22] | Low | No inconsistency | No indirectness | No imprecision | Unlikely | High |
| Age - > 75 years | 55 | | 0.71 [0.35, 1.45] | Low | No inconsistency | No indirectness | No imprecision | Unlikely | High |
| Sex - Female | 138 | | 1.00 [0.60, 1.68] | Low | No inconsistency | No indirectness | No imprecision | Unlikely | High |
| Sex - Male | 273 | | 0.84 [0.61, 1.16] | Low | No inconsistency | No indirectness | No imprecision | Unlikely | High |
| Race - Asia | 727 | | 0.97 [0.84, 1.11] | Low | No inconsistency | No indirectness | No imprecision | Unlikely | High |
| ECOG PS - 0 | 190 | | 0.93 [0.59, 1.46] | Low | No inconsistency | No indirectness | No imprecision | Unlikely | High |
| ECOG PS - 1 | 221 | | 0.86 [0.61, 1.21] | Low | No inconsistency | No indirectness | No imprecision | Unlikely | High |
| Smoking status - Current/former | 296 | | 0.95 [0.69, 1.30] | Low | No inconsistency | No indirectness | No imprecision | Unlikely | High |
| Smoking status - Never | 115 | | 0.70 [0.41, 1.20] | Low | No inconsistency | No indirectness | No imprecision | Unlikely | High |
| Pathological type - Non-squamous | 1529 | | 0.96 [0.87, 1.06] | Low | No inconsistency | No indirectness | No imprecision | Unlikely | High |
| Stage - Stage IV | 1213 | | 0.93 [0.82, 1.06] | Low | No inconsistency | No indirectness | No imprecision | Unlikely | High |
| Brain metastases - Yes | 78 | | 0.93 [0.50, 1.73] | Low | No inconsistency | No indirectness | No imprecision | Unlikely | High |
| Brain metastases - No | 333 | | 0.86 [0.64, 1.16] | Low | No inconsistency | No indirectness | No imprecision | Unlikely | High |
| Liver metastases - Yes | 36 | | 0.91 [0.42, 1.97] | Low | No inconsistency | No indirectness | No imprecision | Unlikely | High |
| Liver metastases - No | 375 | | 0.85 [0.64, 1.13] | Low | No inconsistency | No indirectness | No imprecision | Unlikely | High |
| PD-L1 CPS - <1% | 141 | | 0.87 [0.57, 1.32] | Low | No inconsistency | No indirectness | No imprecision | Unlikely | High |
| PD-L1 CPS - 1%-49% | 107 | | 1.00 [0.55, 1.82] | Low | No inconsistency | No indirectness | No imprecision | Unlikely | High |
| PD-L1 CPS - >50% | 83 | | 1.21 [0.66, 2.23] | Low | No inconsistency | No indirectness | No imprecision | Unlikely | High |
| PD-1/PD-L1 inhibitors type - Atezolizumab | 1213 | | 0.93 [0.82, 1.06] | Low | No inconsistency | No indirectness | No imprecision | Unlikely | High |
| PD-1/PD-L1 inhibitors type - Sintilimab | 316 | | 1.01 [0.86, 1.18] | Low | No inconsistency | No indirectness | No imprecision | Unlikely | High |
| EGFR-mutant - Positive | 440 | | 0.93 [0.82, 1.06] | Low | No inconsistency | No indirectness | No imprecision | Unlikely | High |
| EGFR-mutant - Negative | 287 | | 1.01 [0.86, 1.18] | Low | No inconsistency | No indirectness | No imprecision | Unlikely | High |
| **Subgroup analysis of PFS** |  |  |  |  |  |  |  |  |  |
| Total | 1529 | | 0.76 [0.66, 0.87] | Low | No inconsistency | No indirectness | No imprecision | Unlikely | High |
| Age - < 75 years | 356 | | 0.89 [0.71, 1.12] | Low | No inconsistency | No indirectness | No imprecision | Unlikely | High |
| Age - > 75 years | 55 | | 0.89 [0.51, 1.56] | Low | No inconsistency | No indirectness | No imprecision | Unlikely | High |
| Sex - Female | 138 | | 1.09 [0.76, 1.57] | Low | No inconsistency | No indirectness | No imprecision | Unlikely | High |
| Sex - Male | 273 | | 0.82 [0.63, 1.06] | Low | No inconsistency | No indirectness | No imprecision | Unlikely | High |
| Race - Asia | 727 | | 0.78 [0.67, 0.91] | Low | No inconsistency | No indirectness | No imprecision | Unlikely | High |
| ECOG PS - 0 | 190 | | 0.97 [0.71, 1.32] | Low | No inconsistency | No indirectness | No imprecision | Unlikely | High |
| ECOG PS - 1 | 221 | | 0.84 [0.63, 1.11] | Low | No inconsistency | No indirectness | No imprecision | Unlikely | High |
| Smoking status - Current/former | 296 | | 0.87 [0.68, 1.11] | Low | No inconsistency | No indirectness | No imprecision | Unlikely | High |
| Smoking status - Never | 115 | | 1.01 [0.69, 1.48] | Low | No inconsistency | No indirectness | No imprecision | Unlikely | High |
| Pathological type - Non-squamous | 1529 | | 0.76 [0.66, 0.87] | Low | No inconsistency | No indirectness | No imprecision | Unlikely | High |
| Stage - Stage IV | 1213 | | 0.79 [0.66, 0.94] | Low | No inconsistency | No indirectness | No imprecision | Unlikely | High |
| Brain metastases - Yes | 78 | | 0.77 [0.48, 1.24] | Low | No inconsistency | No indirectness | No imprecision | Unlikely | High |
| Brain metastases - No | 333 | | 0.91 [0.72, 1.15] | Low | No inconsistency | No indirectness | No imprecision | Unlikely | High |
| Liver metastases - Yes | 36 | | 0.40 [0.19, 0.85] | Low | No inconsistency | No indirectness | No imprecision | Unlikely | High |
| Liver metastases - No | 375 | | 0.93 [0.74, 1.16] | Low | No inconsistency | No indirectness | No imprecision | Unlikely | High |
| PD-L1 CPS - <1% | 141 | | 1.11 [0.78, 1.57] | Low | No inconsistency | No indirectness | No imprecision | Unlikely | High |
| PD-L1 CPS - 1%-49% | 107 | | 0.82 [0.54, 1.24] | Low | No inconsistency | No indirectness | No imprecision | Unlikely | High |
| PD-L1 CPS - >50% | 83 | | 0.94 [0.57, 1.55] | Low | No inconsistency | No indirectness | No imprecision | Unlikely | High |
| PD-1/PD-L1 inhibitors type - Atezolizumab | 1213 | | 0.79 [0.66, 0.94] | Low | No inconsistency | No indirectness | No imprecision | Unlikely | High |
| PD-1/PD-L1 inhibitors type - Sintilimab | 316 | | 0.71 [0.57, 0.88] | Low | No inconsistency | No indirectness | No imprecision | Unlikely | High |
| EGFR-mutant - Positive | 440 | | 0.70 [0.58, 0.84] | Low | Serious (-1) | No indirectness | No imprecision | Unlikely | Medium |
| EGFR-mutant - Negative | 287 | | 1.01 [0.78, 1.30] | Low | No inconsistency | No indirectness | No imprecision | Unlikely | High |
| **Responses** |  |  |  |  |  |  |  |  |  |
| ORR | 422/763 | 312/766 | 1.36 [1.22, 1.51] | Low | No inconsistency | No indirectness | No imprecision | Unlikely | High |
| DCR | 471/558 | 446/560 | 1.06 [1.00, 1.12] | Low | No inconsistency | No indirectness | No imprecision | Unlikely | High |
| CR | 13/763 | 9/766 | 1.45 [0.63, 3.37] | Low | No inconsistency | No indirectness | No imprecision | Unlikely | High |
| PR | 409/763 | 303/766 | 1.36 [1.21, 1.51] | Low | No inconsistency | No indirectness | No imprecision | Unlikely | High |
| SD | 169/558 | 225/560 | 0.75 [0.64, 0.89] | Low | No inconsistency | No indirectness | No imprecision | Unlikely | High |
| **Safety** |  |  |  |  |  |  |  |  |  |
| **Safety summary** |  |  |  |  |  |  |  |  |  |
| Total adverse events | 747/763 | 745/766 | 1.01 [0.99, 1.02] | Low | No inconsistency | No indirectness | No imprecision | Unlikely | High |
| Grade 3-5 adverse events | 465/763 | 426/766 | 1.10 [1.01, 1.19] | Low | No inconsistency | No indirectness | No imprecision | Unlikely | High |
| Serious adverse events | 252/558 | 210/560 | 1.29 [0.91, 1.82] | Low | Serious (-1) | No indirectness | No imprecision | Unlikely | Medium |
| Fatal adverse events | 35/558 | 13/560 | 2.70 [1.45, 5.05] | Low | No inconsistency | No indirectness | No imprecision | Unlikely | High |
| Discontinuation due to adverse events | 190/558 | 74/560 | 2.58 [2.03, 3.28] | Low | No inconsistency | No indirectness | No imprecision | Unlikely | High |
| Dose interruption due to adverse events | 256/400 | 209/402 | 1.23 [1.09, 1.39] | Low | No inconsistency | No indirectness | No imprecision | Unlikely | High |
| Treatment-related adverse events | 526/558 | 528/560 | 1.01 [0.96, 1.06] | Low | Serious (-1) | No indirectness | No imprecision | Unlikely | Medium |
| Grade 3-5 treatment-related adverse events | 313/558 | 236/560 | 1.33 [1.18, 1.50] | Low | No inconsistency | No indirectness | No imprecision | Unlikely | High |
| Serious treatment-related adverse events | 155/558 | 114/560 | 1.36 [1.10, 1.69] | Low | No inconsistency | No indirectness | No imprecision | Unlikely | High |
| Fatal treatment-related adverse events | 23/763 | 6/766 | 3.85 [1.58, 9.40] | Low | No inconsistency | No indirectness | No imprecision | Unlikely | High |
| **Any grade adverse Events** |  |  |  |  |  |  |  |  |  |
| Anorexia | 98/205 | 74/206 | 1.33 [1.06, 1.68] | Low | No inconsistency | No indirectness | No imprecision | Unlikely | High |
| Alopecia | 183/400 | 173/402 | 1.06 [0.91, 1.24] | Low | No inconsistency | No indirectness | No imprecision | Unlikely | High |
| Nausea | 320/763 | 268/766 | 1.20 [1.06, 1.36] | Low | No inconsistency | No indirectness | No imprecision | Unlikely | High |
| Malaise | 85/205 | 56/206 | 1.53 [1.16, 2.01] | Low | No inconsistency | No indirectness | No imprecision | Unlikely | High |
| White blood cell count decreased | 146/363 | 168/364 | 0.87 [0.74, 1.02] | Low | No inconsistency | No indirectness | No imprecision | Unlikely | High |
| AST increased | 138/363 | 114/364 | 1.21 [0.99, 1.48] | Low | No inconsistency | No indirectness | No imprecision | Unlikely | High |
| Peripheral neuropathy | 152/400 | 122/402 | 1.25 [1.03, 1.52] | Low | No inconsistency | No indirectness | No imprecision | Unlikely | High |
| Anemia | 269/763 | 284/766 | 0.95 [0.84, 1.08] | Low | No inconsistency | No indirectness | No imprecision | Unlikely | High |
| ALT increased | 126/363 | 109/364 | 1.15 [0.81, 1.64] | Low | Serious (-1) | No indirectness | No imprecision | Unlikely | Medium |
| Fever | 71/205 | 67/206 | 1.06 [0.81, 1.40] | Low | No inconsistency | No indirectness | No imprecision | Unlikely | High |
| Neutrophil count decreased | 250/763 | 232/766 | 1.08 [0.95, 1.23] | Low | No inconsistency | No indirectness | No imprecision | Unlikely | High |
| Decreased appetite | 168/558 | 132/560 | 1.28 [1.06, 1.54] | Low | No inconsistency | No indirectness | No imprecision | Unlikely | High |
| Constipation | 203/763 | 204/766 | 1.01 [0.71, 1.43] | Low | Serious (-1) | No indirectness | No imprecision | Unlikely | Medium |
| Fatigue | 101/400 | 89/402 | 1.14 [0.89, 1.46] | Low | No inconsistency | No indirectness | No imprecision | Unlikely | High |
| Hypertension | 191/763 | 90/766 | 3.06 [0.91, 10.33] | Low | Serious (-1) | No indirectness | No imprecision | Unlikely | Medium |
| Asthenia | 133/558 | 127/560 | 1.05 [0.86, 1.29] | Low | No inconsistency | No indirectness | No imprecision | Unlikely | High |
| Platelet count decreased | 180/763 | 158/766 | 1.14 [0.95, 1.37] | Low | No inconsistency | No indirectness | No imprecision | Unlikely | High |
| Creatinine increased | 73/363 | 52/364 | 1.41 [1.02, 1.94] | Low | No inconsistency | No indirectness | No imprecision | Unlikely | High |
| Proteinuria | 149/763 | 76/766 | 2.31 [0.86, 6.15] | Low | Serious (-1) | No indirectness | No imprecision | Unlikely | Medium |
| Stomatitis | 118/605 | 57/608 | 2.08 [1.56, 2.77] | Low | No inconsistency | No indirectness | No imprecision | Unlikely | High |
| Increased blood thyroid stimulating hormone | 29/158 | 16/158 | 1.81 [1.03, 3.20] | Low | No inconsistency | No indirectness | No imprecision | Unlikely | High |
| Neutropenia | 72/400 | 68/402 | 1.06 [0.79, 1.44] | Low | No inconsistency | No indirectness | No imprecision | Unlikely | High |
| Vomiting | 137/763 | 107/766 | 1.28 [1.02, 1.61] | Low | No inconsistency | No indirectness | No imprecision | Unlikely | High |
| Weight decreased | 28/158 | 25/158 | 1.12 [0.68, 1.83] | Low | No inconsistency | No indirectness | No imprecision | Unlikely | High |
| Epistaxis | 107/605 | 70/608 | 4.38 [0.08, 241.52] | Low | Serious (-1) | No indirectness | No imprecision | Unlikely | Medium |
| Arthralgia | 66/400 | 59/402 | 1.12 [0.81, 1.55] | Low | No inconsistency | No indirectness | No imprecision | Unlikely | High |
| γ-Glutamyltransferase increased | 59/363 | 30/364 | 1.97 [1.31, 2.97] | Low | No inconsistency | No indirectness | No imprecision | Unlikely | High |
| Diarrhea | 123/763 | 91/766 | 1.36 [1.06, 1.74] | Low | No inconsistency | No indirectness | No imprecision | Unlikely | High |
| Hypothyroidism | 24/158 | 17/158 | 1.41 [0.79, 2.52] | Low | No inconsistency | No indirectness | No imprecision | Unlikely | High |
| Dry skin | 31/205 | 18/206 | 1.73 [1.00, 2.99] | Low | No inconsistency | No indirectness | No imprecision | Unlikely | High |
| Hiccups | 31/205 | 27/206 | 1.15 [0.72, 1.86] | Low | No inconsistency | No indirectness | No imprecision | Unlikely | High |
| Increased amylase | 52/363 | 23/364 | 2.27 [1.42, 3.62] | Low | No inconsistency | No indirectness | No imprecision | Unlikely | High |
| Headache | 28/205 | 9/206 | 3.13 [1.51, 6.46] | Low | No inconsistency | No indirectness | No imprecision | Unlikely | High |
| Peripheral edema | 28/205 | 33/206 | 0.85 [0.54, 1.36] | Low | No inconsistency | No indirectness | No imprecision | Unlikely | High |
| Myalgia | 53/400 | 47/402 | 1.13 [0.78, 1.64] | Low | No inconsistency | No indirectness | No imprecision | Unlikely | High |
| Thrombocytopenia | 52/400 | 45/402 | 1.16 [0.80, 1.69] | Low | No inconsistency | No indirectness | No imprecision | Unlikely | High |
| Rash maculopapular | 25/205 | 16/206 | 1.57 [0.86, 2.85] | Low | No inconsistency | No indirectness | No imprecision | Unlikely | High |
| Rash | 92/763 | 66/766 | 1.29 [0.60, 2.78] | Low | Serious (-1) | No indirectness | No imprecision | Unlikely | Medium |
| Insomnia | 23/205 | 33/206 | 0.70 [0.43, 1.15] | Low | No inconsistency | No indirectness | No imprecision | Unlikely | High |
| Back pain | 23/205 | 9/206 | 2.57 [1.22, 5.41] | Low | No inconsistency | No indirectness | No imprecision | Unlikely | High |
| Lymphocyte count decreased | 17/158 | 16/158 | 1.06 [0.56, 2.03] | Low | No inconsistency | No indirectness | No imprecision | Unlikely | High |
| Blood lactate dehydrogenase increase | 17/158 | 18/158 | 0.94 [0.51, 1.76] | Low | No inconsistency | No indirectness | No imprecision | Unlikely | High |
| Paresthesia | 42/400 | 37/402 | 1.14 [0.75, 1.74] | Low | No inconsistency | No indirectness | No imprecision | Unlikely | High |
| Febrile neutropenia | 59/605 | 36/608 | 1.65 [1.11, 2.45] | Low | No inconsistency | No indirectness | No imprecision | Unlikely | High |
| Hyperthyroidism | 15/158 | 17/158 | 0.88 [0.46, 1.70] | Low | No inconsistency | No indirectness | No imprecision | Unlikely | High |
| Pneumonitis | 28/363 | 26/364 | 1.08 [0.65, 1.80] | Low | No inconsistency | No indirectness | No imprecision | Unlikely | High |
| Hypokalaemia | 10/158 | 15/158 | 0.67 [0.31, 1.44] | Low | No inconsistency | No indirectness | No imprecision | Unlikely | High |
| Abnormal liver function | 10/158 | 3/158 | 3.33 [0.93, 11.88] | Low | No inconsistency | No indirectness | No imprecision | Unlikely | High |
| Eczema | 12/205 | 25/206 | 0.48 [0.25, 0.93] | Low | No inconsistency | No indirectness | No imprecision | Unlikely | High |
| Myelosuppression | 5/158 | 1/158 | 5.00 [0.59, 42.31] | Low | No inconsistency | No indirectness | No imprecision | Unlikely | High |
| Pneumonia | 4/158 | 6/158 | 0.67 [0.19, 2.32] | Low | No inconsistency | No indirectness | No imprecision | Unlikely | High |
| Pulmonary embolism | 1/158 | 2/158 | 0.50 [0.05, 5.46] | Low | No inconsistency | No indirectness | No imprecision | Unlikely | High |
| Increased blood triglycerides | 1/158 | 0/158 | 3.00 [0.12, 73.09] | Low | No inconsistency | No indirectness | No imprecision | Unlikely | High |
| Interstitial lung disease | 1/158 | 4/158 | 0.25 [0.03, 2.21] | Low | No inconsistency | No indirectness | No imprecision | Unlikely | High |
| Electrolyte imbalance | 0/158 | 1/158 | 0.33 [0.01, 8.12] | Low | No inconsistency | No indirectness | No imprecision | Unlikely | High |
| Decreased granulocyte count | 0/158 | 2/158 | 0.20 [0.01, 4.13] | Low | No inconsistency | No indirectness | No imprecision | Unlikely | High |
| **Grade 3-5 adverse Events** |  |  |  |  |  |  |  |  |  |
| Anorexia | 74/205 | 12/206 | 6.20 [3.47, 11.05] | Low | No inconsistency | No indirectness | No imprecision | Unlikely | High |
| Neutrophil count decreased | 124/763 | 109/766 | 1.14 [0.91, 1.44] | Low | No inconsistency | No indirectness | No imprecision | Unlikely | High |
| Neutropenia | 54/400 | 44/402 | 1.23 [0.85, 1.79] | Low | No inconsistency | No indirectness | No imprecision | Unlikely | High |
| White blood cell count decreased | 44/363 | 42/364 | 1.05 [0.71, 1.56] | Low | No inconsistency | No indirectness | No imprecision | Unlikely | High |
| Febrile neutropenia | 57/605 | 36/608 | 1.59 [1.06, 2.38] | Low | No inconsistency | No indirectness | No imprecision | Unlikely | High |
| Anemia | 67/763 | 71/766 | 1.01 [0.56, 1.80] | Low | Serious (-1) | No indirectness | No imprecision | Unlikely | Medium |
| Hypertension | 62/763 | 31/766 | 3.16 [0.75, 13.36] | Low | Serious (-1) | No indirectness | No imprecision | Unlikely | Medium |
| Platelet count decreased | 58/763 | 40/766 | 1.46 [0.99, 2.14] | Low | No inconsistency | No indirectness | No imprecision | Unlikely | High |
| ALT increased | 17/363 | 5/364 | 3.20 [1.25, 8.20] | Low | No inconsistency | No indirectness | No imprecision | Unlikely | High |
| Thrombocytopenia | 16/400 | 17/402 | 0.95 [0.48, 1.85] | Low | No inconsistency | No indirectness | No imprecision | Unlikely | High |
| γ-Glutamyltransferase increased | 14/363 | 3/364 | 4.68 [1.36, 16.15] | Low | No inconsistency | No indirectness | No imprecision | Unlikely | High |
| Fatigue | 13/400 | 10/402 | 1.31 [0.58, 2.94] | Low | No inconsistency | No indirectness | No imprecision | Unlikely | High |
| Myelosuppression | 5/158 | 1/158 | 5.00 [0.59, 42.31] | Low | No inconsistency | No indirectness | No imprecision | Unlikely | High |
| Lymphocyte count decreased | 5/158 | 2/158 | 2.50 [0.49, 12.70] | Low | No inconsistency | No indirectness | No imprecision | Unlikely | High |
| Nausea | 24/763 | 18/766 | 1.34 [0.73, 2.45] | Low | No inconsistency | No indirectness | No imprecision | Unlikely | High |
| Decreased appetite | 16/558 | 3/560 | 4.73 [1.50, 14.92] | Low | No inconsistency | No indirectness | No imprecision | Unlikely | High |
| Pneumonitis | 10/363 | 9/364 | 1.12 [0.46, 2.71] | Low | No inconsistency | No indirectness | No imprecision | Unlikely | High |
| Peripheral neuropathy | 11/400 | 9/402 | 1.23 [0.51, 2.93] | Low | No inconsistency | No indirectness | No imprecision | Unlikely | High |
| Increased amylase | 9/363 | 4/364 | 2.26 [0.70, 7.25] | Low | No inconsistency | No indirectness | No imprecision | Unlikely | High |
| Proteinuria | 17/763 | 11/766 | 1.51 [0.73, 3.11] | Low | No inconsistency | No indirectness | No imprecision | Unlikely | High |
| Diarrhea | 15/763 | 6/766 | 2.39 [0.96, 5.95] | Low | No inconsistency | No indirectness | No imprecision | Unlikely | High |
| AST increased | 7/363 | 3/364 | 2.15 [0.61, 7.57] | Low | No inconsistency | No indirectness | No imprecision | Unlikely | High |
| Pneumonia | 3/158 | 1/158 | 3.00 [0.32, 28.53] | Low | No inconsistency | No indirectness | No imprecision | Unlikely | High |
| Hypokalaemia | 3/158 | 2/158 | 1.50 [0.25, 8.86] | Low | No inconsistency | No indirectness | No imprecision | Unlikely | High |
| Asthenia | 10/558 | 13/560 | 0.95 [0.18, 4.95] | Low | Serious (-1) | No indirectness | No imprecision | Unlikely | Medium |
| Vomiting | 13/763 | 7/766 | 1.81 [0.74, 4.38] | Low | No inconsistency | No indirectness | No imprecision | Unlikely | High |
| Rash maculopapular | 3/205 | 1/206 | 3.01 [0.32, 28.74] | Low | No inconsistency | No indirectness | No imprecision | Unlikely | High |
| Peripheral edema | 3/205 | 1/206 | 3.01 [0.32, 28.74] | Low | No inconsistency | No indirectness | No imprecision | Unlikely | High |
| Abnormal liver function | 2/158 | 1/158 | 2.00 [0.18, 21.83] | Low | No inconsistency | No indirectness | No imprecision | Unlikely | High |
| Rash | 8/763 | 4/766 | 1.81 [0.61, 5.37] | Low | No inconsistency | No indirectness | No imprecision | Unlikely | High |
| Malaise | 2/205 | 5/206 | 0.40 [0.08, 2.05] | Low | No inconsistency | No indirectness | No imprecision | Unlikely | High |
| Stomatitis | 5/605 | 2/608 | 2.51 [0.49, 12.90] | Low | No inconsistency | No indirectness | No imprecision | Unlikely | High |
| Creatinine increased | 3/363 | 1/364 | 3.01 [0.32, 28.74] | Low | No inconsistency | No indirectness | No imprecision | Unlikely | High |
| Arthralgia | 3/400 | 4/402 | 0.75 [0.17, 3.35] | Low | No inconsistency | No indirectness | No imprecision | Unlikely | High |
| Epistaxis | 4/605 | 0/608 | 9.04 [0.49, 167.45] | Low | No inconsistency | No indirectness | No imprecision | Unlikely | High |
| Pulmonary embolism | 1/158 | 2/158 | 0.50 [0.05, 5.46] | Low | No inconsistency | No indirectness | No imprecision | Unlikely | High |
| Hyperthyroidism | 1/158 | 0/158 | 3.00 [0.12, 73.09] | Low | No inconsistency | No indirectness | No imprecision | Unlikely | High |
| Myalgia | 2/400 | 1/402 | 2.01 [0.18, 22.08] | Low | No inconsistency | No indirectness | No imprecision | Unlikely | High |
| Fever | 1/205 | 1/206 | 1.00 [0.06, 15.96] | Low | No inconsistency | No indirectness | No imprecision | Unlikely | High |
| Constipation | 0/763 | 2/766 | 0.20 [0.01, 4.16] | Low | No inconsistency | No indirectness | No imprecision | Unlikely | High |
| Decreased granulocyte count | 0/158 | 2/158 | 0.20 [0.01, 4.13] | Low | No inconsistency | No indirectness | No imprecision | Unlikely | High |
| Insomnia | 0/205 | 1/206 | 0.33 [0.01, 8.17] | Low | No inconsistency | No indirectness | No imprecision | Unlikely | High |
| Paresthesia | 0/400 | 1/402 | 0.33 [0.01, 8.20] | Low | No inconsistency | No indirectness | No imprecision | Unlikely | High |
| Interstitial lung disease | 0/158 | 3/158 | 0.14 [0.01, 2.74] | Low | No inconsistency | No indirectness | No imprecision | Unlikely | High |

**Abbreviations:** AE: Adverse Event; ALT: Alanine Aminotransferase; AST: Aspartate Aminotransferase; CI: confidence interval; CR: Complete Response; DCR: Disease Control Rate; GRADE: Grading of Recommendations, Assessment, Development, and Evaluation; HR: Hazard Ratio; ORR: Objective Response Rate; OS: Overall Survival; OSR: Overall Survival Rate; PD-1: programmed cell death protein 1; PD-L1: Programmed cell death 1 ligand 1; PFS: Progression-Free Survival; PFSR: Progression-Free Survival Rate; PIBC: PD-1/PD-L1 Inhibitors plus Bevacizumab plus chemotherapy; PIC: PD-1/PD-L1 Inhibitors plus chemotherapy; PR: Partial response; RCT: Randomized Controlled Trial; RR: Risk Ratio; SD: Stable Disease; TRAEs: Treatment-Related Adverse Events.

^a^ Differences: hazard ratio (HR) for OS and PFS; risk ratios (RR) for OSR, PFSR, adverse events.

^b^ Risk of bias assessed using the Jadad scale for randomized controlled trials.

^c^ Publication bias was explored through visual inspection of the funnel plots.
