# Supplementary material for: PD-1/PD-L1 inhibitors plus bevacizumab plus chemotherapy versus PD-1/PD-L1 inhibitors plus chemotherapy for advanced non-small cell lung cancer: a phase 3 RCT based meta-analysis
Source: Front Oncol. 2025 May 21;15:1496611. doi: 10.3389/fonc.2025.1496611 (PMC12133818; doi:10.3389/fonc.2025.1496611)
Supplement: Supplementary file 11 [file Table5.doc]

**Table S5** Subgroup analysis of overall survival and progression-free survival.

| **Subgroups** | **Overall Survival** | | |  | **Progression-free survival** | | |
| --- | --- | --- | --- | --- | --- | --- | --- |
|  | **Patients** | **HR (95% CI)** | ***P*** |  | **Patients** | **HR (95% CI)** | ***P*** |
| **Total** | 1529 | 0.96 [0.87, 1.06] | 0.43 |  | 1529 | 0.76 [0.66, 0.87] | < 0.0001 |
| **Age** |  |  |  |  |  |  |  |
| < 75 years | 356 | 0.91 [0.68, 1.22] | 0.53 |  | 356 | 0.89 [0.71, 1.12] | 0.32 |
| > 75 years | 55 | 0.71 [0.35, 1.45] | 0.34 |  | 55 | 0.89 [0.51, 1.56] | 0.68 |
| **Sex** |  |  |  |  |  |  |  |
| Female | 138 | 1.00 [0.60, 1.68] | 1 |  | 138 | 1.09 [0.76, 1.57] | 0.64 |
| Male | 273 | 0.84 [0.61, 1.16] | 0.29 |  | 273 | 0.82 [0.63, 1.06] | 0.13 |
| **Race** |  |  |  |  |  |  |  |
| Asia | 727 | 0.97 [0.84, 1.11] | 0.67 |  | 727 | 0.78 [0.67, 0.91] | 0.002 |
| **ECOG PS** |  |  |  |  |  |  |  |
| 0 | 190 | 0.93 [0.59, 1.46] | 0.75 |  | 190 | 0.97 [0.71, 1.32] | 0.85 |
| 1 | 221 | 0.86 [0.61, 1.21] | 0.39 |  | 221 | 0.84 [0.63, 1.11] | 0.23 |
| **Smoking status** |  |  |  |  |  |  |  |
| Current/former | 296 | 0.95 [0.69, 1.30] | 0.75 |  | 296 | 0.87 [0.68, 1.11] | 0.27 |
| Never | 115 | 0.70 [0.41, 1.20] | 0.2 |  | 115 | 1.01 [0.69, 1.48] | 0.96 |
| **Pathological type** |  |  |  |  |  |  |  |
| Non-squamous | 1529 | 0.96 [0.87, 1.06] | 0.43 |  | 1529 | 0.76 [0.66, 0.87] | < 0.0001 |
| **Stage** |  |  |  |  |  |  |  |
| IV | 1213 | 0.93 [0.82, 1.06] | 0.27 |  | 1213 | 0.79 [0.66, 0.94] | 0.007 |
| **Brain metastases** |  |  |  |  |  |  |  |
| Yes | 78 | 0.93 [0.50, 1.73] | 0.82 |  | 78 | 0.77 [0.48, 1.24] | 0.28 |
| No | 333 | 0.86 [0.64, 1.16] | 0.33 |  | 333 | 0.91 [0.72, 1.15] | 0.43 |
| **Liver metastases** |  |  |  |  |  |  |  |
| Yes | 36 | 0.91 [0.42, 1.97] | 0.81 |  | 36 | 0.40 [0.19, 0.85] | 0.02 |
| No | 375 | 0.85 [0.64, 1.13] | 0.27 |  | 375 | 0.93 [0.74, 1.16] | 0.52 |
| **PD-L1 CPS** |  |  |  |  |  |  |  |
| <1% | 141 | 0.87 [0.57, 1.32] | 0.52 |  | 141 | 1.11 [0.78, 1.57] | 0.56 |
| 1%-49% | 107 | 1.00 [0.55, 1.82] | 1 |  | 107 | 0.82 [0.54, 1.24] | 0.35 |
| >50% | 83 | 1.21 [0.66, 2.23] | 0.54 |  | 83 | 0.94 [0.57, 1.55] | 0.81 |
| **PD-1/PD-L1 inhibitors type** |  |  |  |  |  |  |  |
| Atezolizumab | 1213 | 0.93 [0.82, 1.06] | 0.27 |  | 1213 | 0.79 [0.66, 0.94] | 0.007 |
| Sintilimab | 316 | 1.01 [0.86, 1.18] | 0.9 |  | 316 | 0.71 [0.57, 0.88] | 0.002 |
| **EGFR-mutant** |  |  |  |  |  |  |  |
| Positive | 440 | 0.97 [0.83, 1.13] | 0.66 |  | 440 | 0.70 [0.58, 0.84] | 0.0002 |
| Negative | 287 | 1.02 [0.73, 1.42] | 0.91 |  | 287 | 1.01 [0.78, 1.30] | 0.94 |

**Abbreviations:** CI: Confidence interval; CPS: Combined positive score; ECOG PS: Eastern Cooperative Oncology Group Performance Status; EGFR: Epidermal growth factor receptor; HR: Hazard ratio.
