# Supplementary material for: PD-1/PD-L1 inhibitors plus bevacizumab plus chemotherapy versus PD-1/PD-L1 inhibitors plus chemotherapy for advanced non-small cell lung cancer: a phase 3 RCT based meta-analysis
Source: Front Oncol. 2025 May 21;15:1496611. doi: 10.3389/fonc.2025.1496611 (PMC12133818; doi:10.3389/fonc.2025.1496611)
Supplement: Supplementary file 12 [file Table6.doc]

**Table S6** Any grade adverse events (all).

| **Adverse events** | **PIBC** | |  | **PIC** | | **Risk ratio [95% CI]** | **P** |
| --- | --- | --- | --- | --- | --- | --- | --- |
|  | **Event/total** | **%** |  | **Event/total** | **%** |  |  |
| Anorexia | 98/205 | 47.80% |  | 74/206 | 35.92% | 1.33 [1.06, 1.68] | 0.02 |
| Alopecia | 183/400 | 45.75% |  | 173/402 | 43.03% | 1.06 [0.91, 1.24] | 0.44 |
| Nausea | 320/763 | 41.94% |  | 268/766 | 34.99% | 1.20 [1.06, 1.36] | 0.005 |
| Malaise | 85/205 | 41.46% |  | 56/206 | 27.18% | 1.53 [1.16, 2.01] | 0.003 |
| White blood cell count decreased | 146/363 | 40.22% |  | 168/364 | 46.15% | 0.87 [0.74, 1.02] | 0.09 |
| AST increased | 138/363 | 38.02% |  | 114/364 | 31.32% | 1.21 [0.99, 1.48] | 0.06 |
| Peripheral neuropathy | 152/400 | 38.00% |  | 122/402 | 30.35% | 1.25 [1.03, 1.52] | 0.02 |
| Anemia | 269/763 | 35.26% |  | 284/766 | 37.08% | 0.95 [0.84, 1.08] | 0.42 |
| ALT increased | 126/363 | 34.71% |  | 109/364 | 29.95% | 1.15 [0.81, 1.64] | 0.43 |
| Fever | 71/205 | 34.63% |  | 67/206 | 32.52% | 1.06 [0.81, 1.40] | 0.65 |
| Neutrophil count decreased | 250/763 | 32.77% |  | 232/766 | 30.29% | 1.08 [0.95, 1.23] | 0.24 |
| Decreased appetite | 168/558 | 30.11% |  | 132/560 | 23.57% | 1.28 [1.06, 1.54] | 0.01 |
| Constipation | 203/763 | 26.61% |  | 204/766 | 26.63% | 1.01 [0.71, 1.43] | 0.99 |
| Fatigue | 101/400 | 25.25% |  | 89/402 | 22.14% | 1.14 [0.89, 1.46] | 0.3 |
| Hypertension | 191/763 | 25.03% |  | 90/766 | 11.75% | 3.06 [0.91, 10.33] | 0.07 |
| Asthenia | 133/558 | 23.84% |  | 127/560 | 22.68% | 1.05 [0.86, 1.29] | 0.64 |
| Platelet count decreased | 180/763 | 23.59% |  | 158/766 | 20.63% | 1.14 [0.95, 1.37] | 0.15 |
| Creatinine increased | 73/363 | 20.11% |  | 52/364 | 14.29% | 1.41 [1.02, 1.94] | 0.04 |
| Proteinuria | 149/763 | 19.53% |  | 76/766 | 9.92% | 2.31 [0.86, 6.15] | 0.1 |
| Stomatitis | 118/605 | 19.50% |  | 57/608 | 9.38% | 2.08 [1.56, 2.77] | <0.00001 |
| Increased blood thyroid stimulating hormone | 29/158 | 18.35% |  | 16/158 | 10.13% | 1.81 [1.03, 3.20] | 0.04 |
| Neutropenia | 72/400 | 18.00% |  | 68/402 | 16.92% | 1.06 [0.79, 1.44] | 0.69 |
| Vomiting | 137/763 | 17.96% |  | 107/766 | 13.97% | 1.28 [1.02, 1.61] | 0.03 |
| Weight decreased | 28/158 | 17.72% |  | 25/158 | 15.82% | 1.12 [0.68, 1.83] | 0.65 |
| Epistaxis | 107/605 | 17.69% |  | 70/608 | 11.51% | 4.38 [0.08, 241.52] | 0.47 |
| Arthralgia | 66/400 | 16.50% |  | 59/402 | 14.68% | 1.12 [0.81, 1.55] | 0.48 |
| γ-glutamyltransferase increased | 59/363 | 16.25% |  | 30/364 | 8.24% | 1.97 [1.31, 2.97] | 0.001 |
| Diarrhea | 123/763 | 16.12% |  | 91/766 | 11.88% | 1.36 [1.06, 1.74] | 0.02 |
| Hypothyroidism | 24/158 | 15.19% |  | 17/158 | 10.76% | 1.41 [0.79, 2.52] | 0.24 |
| Dry skin | 31/205 | 15.12% |  | 18/206 | 8.74% | 1.73 [1.00, 2.99] | 0.05 |
| Hiccups | 31/205 | 15.12% |  | 27/206 | 13.11% | 1.15 [0.72, 1.86] | 0.56 |
| Increased amylase | 52/363 | 14.33% |  | 23/364 | 6.32% | 2.27 [1.42, 3.62] | 0.0006 |
| Headache | 28/205 | 13.66% |  | 9/206 | 4.37% | 3.13 [1.51, 6.46] | 0.002 |
| Peripheral edema | 28/205 | 13.66% |  | 33/206 | 16.02% | 0.85 [0.54, 1.36] | 0.5 |
| Myalgia | 53/400 | 13.25% |  | 47/402 | 11.69% | 1.13 [0.78, 1.64] | 0.5 |
| Thrombocytopenia | 52/400 | 13.00% |  | 45/402 | 11.19% | 1.16 [0.80, 1.69] | 0.43 |
| Rash maculopapular | 25/205 | 12.20% |  | 16/206 | 7.77% | 1.57 [0.86, 2.85] | 0.14 |
| Rash | 92/763 | 12.06% |  | 66/766 | 8.62% | 1.29 [0.60, 2.78] | 0.52 |
| Insomnia | 23/205 | 11.22% |  | 33/206 | 16.02% | 0.70 [0.43, 1.15] | 0.16 |
| Back pain | 23/205 | 11.22% |  | 9/206 | 4.37% | 2.57 [1.22, 5.41] | 0.01 |
| Lymphocyte count decreased | 17/158 | 10.76% |  | 16/158 | 10.13% | 1.06 [0.56, 2.03] | 0.85 |
| Blood lactate dehydrogenase increased | 17/158 | 10.76% |  | 18/158 | 11.39% | 0.94 [0.51, 1.76] | 0.86 |
| Paresthesia | 42/400 | 10.50% |  | 37/402 | 9.20% | 1.14 [0.75, 1.74] | 0.54 |
| Febrile neutropenia | 59/605 | 9.75% |  | 36/608 | 5.92% | 1.65 [1.11, 2.45] | 0.01 |
| Hyperthyroidism | 15/158 | 9.49% |  | 17/158 | 10.76% | 0.88 [0.46, 1.70] | 0.71 |
| Pneumonitis | 28/363 | 7.71% |  | 26/364 | 7.14% | 1.08 [0.65, 1.80] | 0.77 |
| Hypokalaemia | 10/158 | 6.33% |  | 15/158 | 9.49% | 0.67 [0.31, 1.44] | 0.3 |
| Abnormal liver function | 10/158 | 6.33% |  | 3/158 | 1.90% | 3.33 [0.93, 11.88] | 0.06 |
| Eczema | 12/205 | 5.85% |  | 25/206 | 12.14% | 0.48 [0.25, 0.93] | 0.03 |
| Myelosuppression | 5/158 | 3.16% |  | 1/158 | 0.63% | 5.00 [0.59, 42.31] | 0.14 |
| Pneumonia | 4/158 | 2.53% |  | 6/158 | 3.80% | 0.67 [0.19, 2.32] | 0.52 |
| Pulmonary embolism | 1/158 | 0.63% |  | 2/158 | 1.27% | 0.50 [0.05, 5.46] | 0.57 |
| Increased blood triglycerides | 1/158 | 0.63% |  | 0/158 | 0.00% | 3.00 [0.12, 73.09] | 0.5 |
| Interstitial lung disease | 1/158 | 0.63% |  | 4/158 | 2.53% | 0.25 [0.03, 2.21] | 0.21 |
| Electrolyte imbalance | 0/158 | 0.00% |  | 1/158 | 0.63% | 0.33 [0.01, 8.12] | 0.5 |
| Decreased granulocyte count | 0/158 | 0.00% |  | 2/158 | 1.27% | 0.20 [0.01, 4.13] | 0.3 |

**Abbreviations:** ALT: Alanine Aminotransferase; AST: Aspartate Aminotransferase; CI: confidence interval; PD-1: Programmed cell death protein 1; PD-L1: Programmed cell death 1 ligand 1; PIBC: PD-1/PD-L1 Inhibitors plus Bevacizumab plus chemotherapy; PIC: PD-1/PD-L1 Inhibitors plus chemotherapy.
