# Supplementary material for: PD-1/PD-L1 inhibitors plus bevacizumab plus chemotherapy versus PD-1/PD-L1 inhibitors plus chemotherapy for advanced non-small cell lung cancer: a phase 3 RCT based meta-analysis
Source: Front Oncol. 2025 May 21;15:1496611. doi: 10.3389/fonc.2025.1496611 (PMC12133818; doi:10.3389/fonc.2025.1496611)
Supplement: Supplementary file 13 [file Table7.doc]

**Table S7** Grade 3-5 adverse events (all).

| **Adverse events** | **PIBC** | |  | **PIC** | | **Risk ratio [95% CI]** | **P** |
| --- | --- | --- | --- | --- | --- | --- | --- |
|  | **Event/total** | **%** |  | **Event/total** | **%** |  |  |
| Anorexia | 74/205 | 36.10% |  | 12/206 | 5.83% | 6.20 [3.47, 11.05] | <0.00001 |
| Neutrophil count decreased | 124/763 | 16.25% |  | 109/766 | 14.23% | 1.14 [0.91, 1.44] | 0.26 |
| Neutropenia | 54/400 | 13.50% |  | 44/402 | 10.95% | 1.23 [0.85, 1.79] | 0.27 |
| White blood cell count decreased | 44/363 | 12.12% |  | 42/364 | 0.12 | 1.05 [0.71, 1.56] | 0.81 |
| Febrile neutropenia | 57/605 | 9.42% |  | 36/608 | 5.92% | 1.59 [1.06, 2.38] | 0.02 |
| Anemia | 67/763 | 8.78% |  | 71/766 | 0.09 | 1.01 [0.56, 1.80] | 0.99 |
| Hypertension | 62/763 | 8.13% |  | 31/766 | 4.05% | 3.16 [0.75, 13.36] | 0.12 |
| Platelet count decreased | 58/763 | 7.60% |  | 40/766 | 5.22% | 1.46 [0.99, 2.14] | 0.06 |
| ALT increased | 17/363 | 4.68% |  | 5/364 | 1.37% | 3.20 [1.25, 8.20] | 0.02 |
| Thrombocytopenia | 16/400 | 4.00% |  | 17/402 | 4.23% | 0.95 [0.48, 1.85] | 0.87 |
| γ-glutamyltransferase increased | 14/363 | 3.86% |  | 3/364 | 0.82% | 4.68 [1.36, 16.15] | 0.01 |
| Fatigue | 13/400 | 3.25% |  | 10/402 | 0.02 | 1.31 [0.58, 2.94] | 0.52 |
| Myelosuppression | 5/158 | 3.16% |  | 1/158 | 0.63% | 5.00 [0.59, 42.31] | 0.14 |
| Lymphocyte count decreased | 5/158 | 3.16% |  | 2/158 | 1.27% | 2.50 [0.49, 12.70] | 0.27 |
| Nausea | 24/763 | 3.15% |  | 18/766 | 0.02 | 1.34 [0.73, 2.45] | 0.34 |
| Decreased appetite | 16/558 | 2.87% |  | 3/560 | 0.01 | 4.73 [1.50, 14.92] | 0.01 |
| Pneumonitis | 10/363 | 2.75% |  | 9/364 | 2.47% | 1.12 [0.46, 2.71] | 0.81 |
| Peripheral neuropathy | 11/400 | 2.75% |  | 9/402 | 2.24% | 1.23 [0.51, 2.93] | 0.64 |
| Increased amylase | 9/363 | 2.48% |  | 4/364 | 1.10% | 2.26 [0.70, 7.25] | 0.17 |
| Proteinuria | 17/763 | 2.23% |  | 11/766 | 1.44% | 1.51 [0.73, 3.11] | 0.27 |
| Diarrhea | 15/763 | 1.97% |  | 6/766 | 0.78% | 2.39 [0.96, 5.95] | 0.06 |
| AST increased | 7/363 | 1.93% |  | 3/364 | 0.01 | 2.15 [0.61, 7.57] | 0.23 |
| Pneumonia | 3/158 | 1.90% |  | 1/158 | 0.01 | 3.00 [0.32, 28.53] | 0.34 |
| Hypokalaemia | 3/158 | 1.90% |  | 2/158 | 1.27% | 1.50 [0.25, 8.86] | 0.65 |
| Asthenia | 10/558 | 1.79% |  | 13/560 | 2.32% | 0.95 [0.18, 4.95] | 0.95 |
| Vomiting | 13/763 | 1.70% |  | 7/766 | 0.91% | 1.81 [0.74, 4.38] | 0.19 |
| Rash maculopapular | 3/205 | 1.46% |  | 1/206 | 0.49% | 3.01 [0.32, 28.74] | 0.34 |
| Peripheral edema | 3/205 | 1.46% |  | 1/206 | 0.49% | 3.01 [0.32, 28.74] | 0.34 |
| Abnormal liver function | 2/158 | 1.27% |  | 1/158 | 0.63% | 2.00 [0.18, 21.83] | 0.57 |
| Rash | 8/763 | 1.05% |  | 4/766 | 0.52% | 1.81 [0.61, 5.37] | 0.29 |
| Malaise | 2/205 | 0.98% |  | 5/206 | 2.43% | 0.40 [0.08, 2.05] | 0.27 |
| Stomatitis | 5/605 | 0.83% |  | 2/608 | 0.33% | 2.51 [0.49, 12.90] | 0.27 |
| Creatinine increased | 3/363 | 0.83% |  | 1/364 | 0.27% | 3.01 [0.32, 28.74] | 0.34 |
| Arthralgia | 3/400 | 0.75% |  | 4/402 | 0.01 | 0.75 [0.17, 3.35] | 0.71 |
| Epistaxis | 4/605 | 0.66% |  | 0/608 | 0.00% | 9.04 [0.49, 167.45] | 0.14 |
| Pulmonary embolism | 1/158 | 0.63% |  | 2/158 | 0.01 | 0.50 [0.05, 5.46] | 0.57 |
| Hyperthyroidism | 1/158 | 0.63% |  | 0/158 | 0.00% | 3.00 [0.12, 73.09] | 0.50 |
| Myalgia | 2/400 | 0.50% |  | 1/402 | 0.25% | 2.01 [0.18, 22.08] | 0.57 |
| Fever | 1/205 | 0.49% |  | 1/206 | 0.49% | 1.00 [0.06, 15.96] | 1.00 |
| Constipation | 0/763 | 0.00% |  | 2/766 | 0.26% | 0.20 [0.01, 4.16] | 0.30 |
| Decreased granulocyte count | 0/158 | 0.00% |  | 2/158 | 1.27% | 0.20 [0.01, 4.13] | 0.30 |
| Insomnia | 0/205 | 0.00% |  | 1/206 | 0.49% | 0.33 [0.01, 8.17] | 0.50 |
| Paresthesia | 0/400 | 0.00% |  | 1/402 | 0.00 | 0.33 [0.01, 8.20] | 0.50 |
| Interstitial lung disease | 0/158 | 0.00% |  | 3/158 | 1.90% | 0.14 [0.01, 2.74] | 0.20 |

**Abbreviations:** ALT: Alanine Aminotransferase; AST: Aspartate Aminotransferase; CI: confidence interval; PD-1: Programmed cell death protein 1; PD-L1: Programmed cell death 1 ligand 1; PIBC: PD-1/PD-L1 Inhibitors plus Bevacizumab plus chemotherapy; PIC: PD-1/PD-L1 Inhibitors plus chemotherapy.
