# Supplementary material for: PD-1/PD-L1 inhibitors plus bevacizumab plus chemotherapy versus PD-1/PD-L1 inhibitors plus chemotherapy for advanced non-small cell lung cancer: a phase 3 RCT based meta-analysis
Source: Front Oncol. 2025 May 21;15:1496611. doi: 10.3389/fonc.2025.1496611 (PMC12133818; doi:10.3389/fonc.2025.1496611)
Supplement: Supplementary file 14 [file Table8.doc]

**Table S8** Data availability of included studies.

| **Included studies** | **Digital object identifier** | **PubMed IDs** |
| --- | --- | --- |
| **IMpower150 (NCT02366143)** |  |  |
| Nogami 2022[15] | 10.1136/jitc-2021-003027 | 35190375 |
| West 2022[16] | 10.1016/j.jtho.2021.09.014 | 34626838 |
| Socinski 2021[17] | 10.1016/j.jtho.2021.07.009 | 34311108 |
| Reck 2020[18] | 10.1200/JCO.19.03158 | 32459597 |
| Reck 2019[19] | 10.1016/S2213-2600(19)30084-0 | 30922878 |
| Socinski 2018[9] | 10.1056/NEJMoa1716948 | 29863955 |
| **jRCT2080224500** |  |  |
| Shiraishi 2024[10] | 10.1001/jamaoncol.2023.5258 | 38127362 |
| **ORIENT-31 (NCT03802240)** |  |  |
| Lu 2023[20] | 10.1016/S2213-2600(23)00135-2 | 37156249 |
| Lu 2022[11] | 10.1016/S1470-2045(22)00382-5 | 35908558 |
